# Supplementary material for: ProFertil study protocol for the investigation of gonadotropin-releasing hormone agonists (GnRHa) during chemotherapy aiming at fertility protection of young women and teenagers with cancer in Sweden—a phase III randomised double-blinded placebo-controlled study
Source: BMJ Open. 2023 Dec 8;13(12):e078023. doi: 10.1136/bmjopen-2023-078023 (PMC10728964; doi:10.1136/bmjopen-2023-078023)
Supplement: Supplementary data [file bmjopen-2023-078023supp001.pdf]

CLINICAL STUDY PROTOCOL

A Phase III Randomized Double-Blinded Placebo-Controlled Study of Use of GnRHa during Chemotherapy for Fertility Protection of Young Women and Teenagers with Cancer - ProFertil

Study code:	ProFertil
EudraCT number:	2020-004780-71
Version number:	2.5
Date:	2022-09-27

Sponsor/Principal Investigator: Kenny A. Rodriguez-Wallberg, Prof., M.D., Ph.D.
MU Gynecology and Reproductive Medicine
Women's Health and Professional Health Theme
Karolinska University Hospital

Study Code: ProFertil
Version No: 2.5
Date: 2022-09-27
EudraCT No: 2020-004780-71

Table of contents

Signature page 6
Contact information 7
List of used acronymes and abbreviations 9
1. Synopsis 11
2. Background and rationale 15
2.1. Ovarian protection 15
2.2. Pre-clinical and clinical studies for ovarian protection 15
2.3. Study rationale 18
2.4. Study treatment 19
2.5. Chemotherapy for breast cancer 20
2.6. Chemotherapy for acute leukemias 22
2.7. Chemotherapy for lymphomas 23
2.8. Chemotherapy for sarcomas 24
3. Risk-benefit evaluation 24
3.1. Risks 24
3.2. Benefits 24
3.3. Risk-Benefit Assessment 25
4. Study objectives 25
4.1. Primary objective 26
4.2. Secondary objectives 26
4.3. Primary endpoint 27
4.4. Secondary endpoints 27
5. Study design and procedures 29
5.1. Overall study design 29
5.2. Procedures and flow chart 29
5.2.1. Assessments and procedures 29
5.2.2. Study visits 31
5.3. Biological sampling procedures 34
5.3.1. Handling, storage, and destruction of biological samples 34
5.3.2. Total volume of blood per study subject 34

5.3.3. Biobank ... 34
5.4. End of Study ... 34
6. Subject selection ... 34
6.1. Inclusion criteria ... 34
6.2. Exclusion criteria ... 35
6.3. Screening ... 35
6.4. Withdrawal criteria ... 36
7. Study treatments ... 36
7.1. Description of investigational product(s) ... 36
7.2. Dose and administration ... 37
7.3. Packaging, labelling, and handling of investigational products(s) ... 38
7.4. Drug accountability and treatment compliance ... 39
7.5. Randomization ... 39
7.6. Blinding ... 40
7.7. Code breaking ... 40
7.8. Concomitant medications ... 40
7.9. Destruction ... 40
7.10. Treatment after study end ... 40
8. Assessment of efficacy and safety ... 41
8.1. Assessment of clinical efficacy ... 41
8.1.1. Primary variable ... 42
8.1.2. Secondary variable(s) ... 42
8.2. Assessment of clinical safety ... 42
9. Handling of Adverse Events ... 43
9.1. Definitions ... 43
9.1.1. Adverse Event (AE) ... 43
9.1.2. Adverse Drug Reaction (ADR) ... 43
9.1.3. Serious Adverse Event (SAE) ... 43
9.1.4. Suspected Unexpected Serious Adverse Reaction (SUSAR) ... 43
9.2. Assessment of adverse events ... 43
9.2.1. Assessment of causal relationship ... 43
9.2.2. Assessment of severity ... 44

9.3. Reporting and registration of adverse events 44
9.3.1. Reporting of adverse events (AE) 46
9.3.2. Reporting of serious adverse events (SAE) 46
9.3.3. Reporting of Suspected Unexpected Serious Adverse Reactions (SUSAR) 46
9.4. Follow-up of Adverse Events 47
9.5. Annual Safety Report (Development Safety Update Report, DSUR) 47
9.6. Procedures in case of emergencies, overdose or pregnancy 47
9.7. Reference Safety Information 47
10. Statistics 47
10.1. Statistical analysis plan and database locks 47
10.2. Analysis population 47
10.3. Method of statistical analyses 48
10.3.1. General Principles 48
10.3.2. Primary and secondary analyses 48
10.3.3. Handling of missing data 49
10.3.4. Subgroup analysis 49
10.4. Adjustment of significance and confidence interval 49
10.5. Sample size calculations 50
10.6. Interim report 50
11. Quality Control and Quality Assurance 50
11.1. Training of Study Personnel 50
11.2. Monitoring 51
11.3. Source data 51
11.4. Deviations or serious breaches 52
11.5. Audits and inspections 52
12. Ethics 52
12.1. Compliance to the protocol, GCP and regulations 52
12.2. Ethical review of the study 52
12.3. Procedure for obtaining informed consent 52
12.4. Data protection 53
12.5. Insurances 53
13. Substantial changes to the study 53

14. Collection, handling, and archiving data 54
14.1. Subject Identification Log 54
14.2. Case Report Form 54
14.3. Archiving 54
15. Notification of study completion, reporting, and publication 55
16. References 55

Study Code: ProFertil
Version No: 2.5
Date: 2022-09-27
EudraCT No: 2020-004780-71

Signature page

Sponsor
I am responsible for ensuring that this protocol includes all essential information to be able to conduct this study. I will submit the protocol and all other important study-related information to the responsible investigator(s) so that they can conduct the study correctly. I am aware that it is my responsibility to hold the staff members who work with this study informed and trained.

[signature] 22-10-12

Sponsor's signature Date

Kenny Rodriguez-Wallberg

Printed name

Principal Investigator
I have read this protocol and it contains all essential information to conduct this study. By signing my name below, I agree to conduct the study in compliance with this protocol, the Declaration of Helsinki, ICH GCP (Good Clinical Practice) guidelines and the national and international regulations governing the conduct of this clinical study.

I will submit this protocol and all other important study-related information to the staff members and responsible investigators who participate in this study, so that they can conduct the study correctly. I am aware of my responsibility to continuously keep the staff members and responsible investigators who work with this study informed and trained.

I am aware that quality control of this study will be performed in the form of monitoring, possibly audit, and possibly inspection.

Principal Investigator's signature Date

Printed name

Study Code: ProFertil
Version No: 2.5
Date: 2022-09-27
EudraCT No: 2020-004780-71

Contact information

**Role**	
Sponsor	Karolinska University Hospital<br>Women's Health and Professional Health Theme<br>MU Gynecology and Reproductive Medicine<br>SE-171 76 Stockholm<br>Sweden
Sponsor representative	Kenny A. Rodriguez-Wallberg, Prof., M.D., Ph.D.<br>MU Gynecology and Reproductive Medicine<br>Women's Health and Professional Health Theme<br>Karolinska University Hospital<br>+46 858580000<br>kenny.rodriguez-wallberg@ki.se
Statistical Analyses	Daniel Bruce<br>SDS Life Science<br>Svärdvägen 23<br>SE-182 33 Danderyd<br>daniel.bruce@sdslifescience.com<br><br>Sandra Eloranta<br>Dept. of Medicine<br>Clinical Epidemiology<br>Karolinska Institutet<br>SE-171 64 Solna<br>sandra.eloranta@ki.se
Project Manager	Hanna Nilsson<br>Dept. of Oncology-Pathology<br>Karolinska Institutet<br>SE-171 64 Solna<br>hanna.nilsson@ki.se
Clinical Research Manager / Clinical Research Associate	Réka Conrad<br>Clinical Trial Office<br>MU Center for Clinical Cancer Studies<br>Cancer Theme<br>Karolinska University Hospital<br>SE-171 76 Stockholm<br>reka.conrad@regionstockholm.se
Data Manager	Mats Hellström<br>Clinical Trial Office<br>MU Center for Clinical Cancer Studies<br>Cancer Theme<br>Karolinska University Hospital

**Role**	
	SE-171 76 Stockholm<br>mats.hellstrom@regionstockholm.se

Study Code: ProFertil
Version No: 2.5
Date: 2022-09-27
EudraCT No: 2020-004780-71

List of used acronymes and abbreviations

Abbreviation	Term/Explanation
ABVD	Adriamycin (Doxorubicin), Bleomycin, Vinblastine, Dacarbazine
AE	Adverse Event = any untoward medical occurrence
AIDA	Acute Lymphatic Leukemia-Trans Retinoic Acid, Idarubicin
ALCL	Anaplastic Large Cell Lymphoma
ALL	Acute Lymphatic Leukemia
AMH	Anti-Müllerian Hormone
AML	Acute Myeloid Leukemia
ANCOVA	Analysis of Covariance
AR	Adverse Reaction = adverse event, that is each unfavorable and unexpected reaction to a study treatment, regardless of dose
ASCO	American Society of Clinical Oncology
BEACOPP	Bleomycin, Etoposide, Adriamycin (Doxorubicin), Cyclopshosphamide, Oncovin (Vincristine), Procarbazine, Prednisone
B-NHL	B-cell Non-Hodgkin Lymphoma
ECOG	Eastern Cooperative Oncology Group
R-CHOP	Rituximab, Cyclophosphamide, Adriamycin (Doxorubicin), Oncovin (Vincristine), Prednisone
R-CHOEP	Rituximab, Cyclophosphamide, Adriamycin (Doxorubicin), Oncovin (Vincristine), Etoposide, Prednisone
CPA	Cyclophosphamide
CRF	Case Report Form
CWS	Cooperative Weichteilsarkom Studiengruppe
DBH	Dutch-Belgian-Holland
DSUR	Development Safety Update Report = annual safety report
EC	Epirubicin, Cyclophosphamide
EoT	End of Treatment, in this study end of gonadotoxic chemotherapy
EPM	Etikprövningsmyndigheten (English: Swedish Ethical Review Authority)
EURAMOS	European and American Osteosarcoma Study
EuroNetPHL	European Network Pediatric Hodgkin Lymphoma
FEC	Fluorouracil, Epirubicin, Cyclophosphamide
FSH	Follicle Stimulating Hormone
GCP	Good Clinical Practice
GnRHa	Gonadotropin-Releasing Hormone Agonists
HR	High Risk
ICH	International Council for Harmonization
IR	Intermediate Risk
ITT	Intention-to-treat = including all data from all subjects who have participated in the study
LBL	Lymphoblastic Lymphoma
LH	Luteinizing Hormone

LVFS	Läkemedelsverkets författningssamling (English: Swedish Medical Products Agency's statutes)
MMRM	Mixed Model Repeated Measures
NHL	Non-Hodgkin Lymphoma
NOPHO	Nordic Society for Pediatric Haematology and Oncology
NOPHO-DBH-AML	Nordic Society for Pediatric Haematology and Oncology-Dutch-Belgian-Holland-Acute Myeloid Leukemia
PP	Per Protocol Analysis = including only data from subjects who have completed the study completely in accordance with the protocol, with no deviations from the protocol
SAE	Serious Adverse Event = serious untoward medical occurrence
SCT	Stem Cell Transplantation
SPC or SmPC	Summary of Product Characteristics
SR	Standard Risk
SUSAR	Suspected Unexpected Serious Adverse Reaction
TBI	Total Body Irradiation
TC	Taxotere, Cyclosphosphamide
TL	Treatment Level/Transformed non-Hodgkin Lymphoma

Study Code: ProFertil
Version No: 2.5
Date: 2022-09-27
EudraCT No: 2020-004780-71

1. Synopsis

EudraCT number: 2020-004780-71

Title: A Phase III randomized double-blinded, placebo-controlled study of use of GnRHa during chemotherapy for fertility protection of young women (age 18-42) and teenagers (age 14-17) with cancer – ProFertil

Study code: ProFertil

Short background/Rationale/Purpose:
The aim of the study is to investigate if the administration of Gonadotropin-Releasing Hormone agonist (GnRHa) during cancer treatment can preserve the fertility of young female cancer subjects.

Primary objective:
To estimate the changes in ovarian reserve following chemotherapy for treatment of cancer with or without GnRHa by determination of the Anti-Müllerian hormone (AMH) at 12 months after end of gonadotoxic treatment (EoT) in women with breast cancer.

Secondary objectives:
Key secondary objective:
To estimate the changes in ovarian reserve following chemotherapy for treatment of cancer with or without GnRHa by determination of the AMH at 12 months after EoT in women with acute leukemias, lymphomas and sarcomas.

Secondary objectives (to be evaluated at EoT, 6, 12 months after EoT and continuously during follow-up years 2, 3, 4, 5 after EoT):

- To estimate the changes in ovarian reserve with or without GnRHa by determination of the antral follicle counts (AFC) in women with breast cancer
- To estimate the changes in ovarian reserve with or without GnRHa by determination of the AFC in women with acute leukemias, lymphomas and sarcomas
- To estimate the changes in ovarian reserve with or without GnRHa by longitudinal observation of AMH levels in women with breast cancer
- To estimate the changes in ovarian reserve with or without GnRHa by longitudinal observation of AMH levels in women with acute leukemias, lymphomas and sarcomas
- To compare the proportion of females with or without GnRHa that develop ovarian insufficiency by determination of follicle stimulating hormone (FSH), inhibin and estradiol at standardized timepoints
- To investigate the impact of body mass index (BMI), use of contraceptives and endocrine adjuvant therapy in changes of ovarian reserve with or without GnRHa by longitudinal observation of AMH levels, FSH, inhibin and estradiol at standardized timepoints
- To investigate the effect of GnRHa with or without GnRHa on ovarian blood supply
- To estimate the proportion of females with or without GnRHa that develop amenorrhea (no menstruations)
- To investigate fertility and childbirth after cancer treatment in women with or without GnRHa who attempt pregnancy during follow-up

- To determine health-related quality of life (QoL) with or without GnRHa during chemotherapy and after cancer treatment
- To study development of co-morbidities during follow-up and bone mineral density after completion of cancer treatment with or without GnRHa
- To estimate disease-specific oncologic outcomes, recurrence rate, overall survival and disease-free survival with or without GnRHa

Study design:
Prospective, randomized, double-blinded, placebo-controlled, phase III study

Study population:
Adult women and teenagers diagnosed with breast cancer, acute leukemias, lymphomas (Hodgkin and non-Hodgkin) or sarcomas (osteo, soft tissue and Ewing) treated with chemotherapy

Number of subjects:
The study will include approximately 300 subjects with breast cancer.
In addition, up to 200 subjects with lymphoma, acute leukemias and sarcomas will be recruited.
Subjects will be randomized 1:1 to treatment with GnRHa (triptorelin) or placebo.

Inclusion criteria:
- Signed informed consent
- Age 14-42 years at cancer diagnosis
- Female subjects with breast cancer or acute leukemias, lymphomas (Hodgkin and non-Hodgkin) or sarcomas (osteo, soft tissue and Ewing) confirmed by histology and assigned for disease-specific chemotherapy
- Confirmed menarche
- ECOG performance status 0-1
- Adequate bone marrow, renal, hepatic and cardiac functions and absence of other uncontrolled medical or psychiatric disorders

Exclusion criteria:
Subjects must not be included in the study if any of the following criteria are met:
- Ongoing treatment with GnRHa at baseline
- Demonstrated premature ovarian failure at time of randomization according to clinical or biochemical data
- Previous or planned bilateral oophorectomy
- Pregnancy or breastfeeding at time of start of chemotherapy
- Other malignancy diagnosed within the last five years
- Uncontrolled hypertension, heart, liver, kidney related or other uncontrolled medical or psychiatric disorders including previous or current diagnosis of anorexia
- Known osteoporosis
- Known refractory thrombocytopenia in subjects with acute leukemias
- Known or suspected allergy against triptorelin
- Direct radiation of the gonads previous or planned (total body irradiation allowed)
- Mental inability, reluctance or language difficulties that result in difficulty understanding the meaning of study participation

Investigational product(s), dosage, administration:
3.75 mg (1-month' effect) or 11.25 mg (3-months' effect) of triptorelin administered as intramuscular injection until the completion of cytostatic treatment with a potential gonadotoxic effect

Primary endpoint:
The difference in recovery of AMH levels at follow-up 12 months after EoT, relative to AMH levels at EoT, as compared between the GnRHa group and the placebo group in women with breast cancer.

Secondary endpoints:
Key secondary endpoint:
The difference in recovery of AMH levels at follow-up 12 months after EoT, relative to AMH levels at EoT, between the GnRHa group and the placebo group in women with acute leukemias, lymphomas and sarcomas.

Secondary endpoints:

- Comparison of AFC measured by ultrasound at EoT, 6, 12 months after EoT and continuously during follow-up years 2, 3, 4, 5 after EoT, between the GnRHa group and the placebo group:
  - In women with breast cancer
  - In women with acute leukemias, lymphomas and sarcomas
- The difference in recovery of AMH levels at 6 months, and follow-up years 2, 3, 4, 5 after EoT, between the GnRHa group and the placebo group:
  - In women with breast cancer
  - In women with acute leukemias, lymphomas and sarcomas
- Comparison of FSH, inhibin and estradiol performed at EoT, 6, 12 months after EoT and continuously during follow-up years 2, 3, 4, 5 after EoT, between the GnRHa group and the placebo group:
  - In women with breast cancer
  - In women with acute leukemias, lymphomas and sarcomas
- Comparison of blood flow to the ovarian artery (right and left Doppler flow PI, RI) at baseline, EoT, 6, 12 months after EoT and continuously during follow-up years 2, 3, 4, 5 after EoT, between the GnRHa group and the placebo group:
  - In women with breast cancer
  - In women with acute leukemias, lymphomas and sarcomas
- Comparison of the proportion that develop amenorrhea (no menstruations) at EoT, 6, 12 months after EoT and continuously during follow-up years 2, 3, 4, 5 after EoT, between the GnRHa group and the placebo group:
  - In women with breast cancer
  - In women with acute leukemias, lymphomas and sarcomas
- Investigation of the impact of BMI, use of contraceptives and endocrine adjuvant therapy in changes of ovarian reserve with or without GnRHa by longitudinal observation of AMH levels, FSH, inhibin and estradiol at standardized timepoints
- Comparison of of pregnancy wish, pregnancy attempts and pregnancy outcomes at EoT, 6, 12 months after EoT and continuously during follow-up years 2, 3, 4, 5 after EoT, between the GnRHa group and the placebo group:

- In women with breast cancer
- In women with acute leukemias, lymphomas and sarcomas

- Comparison of health-related QoL, sexuality and reproductive health examined at EoT, 6, 12 months after EoT and continuously during follow-up years 2, 3, 4, 5 after EoT, between the GnRHa group and the placebo group:
  - In women with breast cancer
  - In women with acute leukemias, lymphomas and sarcomas
- Comparison of bone mineral density at baseline, EoT and 12 months after EoT and follow-up year 5, between the GnRHa group and the placebo group:
  - In women with breast cancer
  - In women with acute leukemias, lymphomas and sarcomas
- Investigation of recurrence rate, overall survival and disease-free survival at 12 months after EoT and follow-up years 2, 3, 4, 5 after EoT, between the GnRHa group and the placebo group:
  - In women with breast cancer
  - In women with acute leukemias, lymphomas and sarcomas

Statistical methods:
The primary endpoint will be analysed using an analysis of covariance model (ANCOVA). The model will include treatment group, age, ovarian reserve (AMH) at baseline, inclusion site and type of cancer treatment as covariates. If assumptions underlying the ANCOVA are not fulfilled a non-parametric analysis will be used instead.
All statistical tests will be performed at a two-sided significance level of $\alpha = 0.05$.

Determination of sample size:
The sample size calculation was done assuming a mean difference in $\log_{10}$-transformed AMH-levels of 0.22 between GnRHa group and placebo group (with a ratio 1:1). It was further assumed that data are approximately normally distributed, and that each treatment group has a standard deviation of 0.63. Under these assumptions, the hypothesis test (a two-sided test with significance level 5 %) of the mean effect between GnRHa group vs placebo group will have a power of at least 80% if the sample size is 300 (including an assumed 15% drop-out rate). The sample size calculation was based on Goldfarb et al. (2021).

Study period: Q2 2022-Q2 2030

2. Background and rationale

2.1. Ovarian protection

Much controversy still exists on the use of a gonadotropin-releasing hormone agonists (GnRHa) co-treatment aiming at suppressing the ovarian function transiently during chemotherapy, with the final objective to prevent ovarian damage and preserve female fertility through protection of the ovarian reserve (Chapman and Sutcliffe, 1981).

Physiologically, primordial follicles, which make up the ovarian reserve, are non-proliferating, lack both follicle stimulating hormone (FSH) and GnRH receptors and their initial recruitment is not controlled by gonadotropins (Oktay, Briggs and Gosden, 1997), thus the proposed initial mechanism for GnRHa protection does not seem plausible. Therefore, it is not likely that suppressing FSH and luteinizing hormone (LH) secretion will reduce the impact of chemotherapy on primordial follicles by keeping them dormant (Oktay, Rodriguez-Wallberg & Munster 2015; Rodriguez-Wallberg et al, 2016, Turan et al, 2019). The American Society of Clinical Oncology (ASCO)'s expert panel did not recommend ovarian suppression for fertility preservation in its recent update of the fertility preservation guidelines published in July 2018 (Oktay et al, 2018). However, several medical societies are currently advocating its utilization for preservation of fertility, although the efficacy of GnRHa co-treatment is unproven. A recent scientific debate article has highlighted the current inconsistencies among research publications (Dolmans et al., 2020).

The current situation worldwide is that GnRHa use is becoming widely utilized with the hope to protect the ovaries from chemotherapy, even in subjects undergoing less gonadotoxic treatments, such as those indicated for benign diseases. In most countries, the GnRHa treatment is offered because it is relatively inexpensive, in comparison to the higher costs of proven methods for fertility preservation such as the cryopreservation of eggs, embryos or ovarian tissue (Oktay et al, 2018). In Sweden, the healthcare services available to the whole population includes the performance of assisted reproductive technologies aimed at fertility preservation when medically indicated, and thus subjects may undergo treatments aimed at cryopreservation of eggs, embryos or ovarian tissue within the tax-funded healthcare. A prospective Swedish cohort study of >1200 women and girls recently published indicates that the procedures needed to obtain eggs or ovarian tissue for fertility preservation of female subjects are both safe and efficacious (Rodriguez-Wallberg et al, 2019a).

Regarding the use of GnRHa co-treatment aimed at fertility preservation, no specific studies have been performed for the investigation of its long-term effects. In a recent meta-analysis, GnRHa co-treatment given during chemotherapy was judged as not dangerous (Lambertini et al, 2018), nevertheless the GnRHa preparations resulted in some adverse effects that impaired cancer subjects' quality of life. In Sweden, several medical oncologists have started to offer GnRHa co-treatment to young female subjects undergoing treatment for cancer (data communicated verbally during the Nordic Society for Paediatric Haematology and Oncology (NOPHO), Stockholm May 20th, 2017) and a few Swedish groups responsible for cancer healthcare programs have initiated discussions on revising the current recommendations to eventually add the use of GnRHa co-treatment when treating young women for cancer.

2.2. Pre-clinical and clinical studies for ovarian protection

Data from animal experiments on GnRHa have shown conflictive results (Table 1).

Table 1. Data from animal experiments investigating GnRHa co-treatment during gonadotoxic treatment

Animal model	Author, year	Gonadotoxic treatment	Protective effect (YES/NO)	Controlled study
Rat	*Ataya, 1985*	chemotherapy	**YES**	yes
	Montz, 1991	chemotherapy	**YES**	yes
	Jarrell, 1987	chemotherapy	**NO**	yes
	Bokser, 1990	chemotherapy	**NO**	yes
	Meistrich, 1994	chemotherapy	**NO**	yes
Mouse	*Meirow, 2004*	chemotherapy	**YES**	yes
	Danforth, 2005	chemotherapy	**YES**	yes
	Yuce, 2004	chemotherapy	**NO**	yes
	Use of GnRH antagonist			
	Demeestere 2018	Both in vivo and in vitro chemotherapy	**NO**	yes
Monkey	*Ataya, 1995*	chemotherapy	**YES**	No
	Ataya 1995	radiotherapy	**NO**	No

With regards to clinical data, studies supporting a beneficial effect of GnRHa co-treatment on ovarian function have been small, mainly retrospective and uncontrolled, and have used resumption of menstruations as surrogate to ovarian function after chemotherapy. An important development for the clinical estimation of the female ovarian reserve has been the establishment of laboratory methods for accurately determining serum concentration of Anti-Müllerian Hormone (AMH). This hormone is secreted by the cohort of growing ovarian follicles and it has been correlated with the number of primordial follicles that constitute the female ovarian pool, as well as with the number of antral follicles that can be recruited in one menstrual cycle. The AMH also correlates with the response to hormonal stimulation and the number of eggs obtained for treatment with assisted reproductive techniques (Dewailly et al., 2015). The serum AMH concentration is currently considered the gold standard marker of ovarian reserve and since 2010 it has been included as outcome measure in several of the randomized studies available on GnRHa co-treatment. Importantly, none of the studies investigating changes in ovarian markers following chemotherapy have found a beneficial effect of GnRHa co-treatment. Randomized studies are shown in Table 2.

Table 2. Data on biochemical markers of ovarian reserve from randomized controlled studies investigating GnRHa co-treatment during gonadotoxic treatment in women with cancer.

Type of cancer	Author, year	Ovarian marker studied	Protective effect (Yes/No)	Study results
Hodgkin lymphoma	*Nitzschke, 2010*	AMH* and Inhibin	**No**	Follow-up 2.7 years<br>No difference between the groups
Hodgkin lymphoma	*Boehringer 2010*	AMH* and Inhibin	**No**	Follow-up 18 months<br>Ovarian markers reduced in both arms
Breast cancer	*Munster 2010*	Inhibin	**No**	Follow-up 18 months Two pregnancies in the control group
Breast cancer	*Gerber, 2011*	AMH* and Inhibin	**No**	Follow-up 6 months<br>Ovarian markers reduced in both arms
Breast cancer	*Elgindy. 2011*	AMH*	**No**	Follow-up 12 months<br>Ovarian marker reduced in both groups

Breast cancer	*Del Mastro, 2011*	No sensitive ovarian markers investigated. FSH was randomly assessed	**Yes?**	Menstruation resumption reported but subjectively in an unblinded study
Breast cancer	*Moore, 2015*	No sensitive ovarian markers investigated, FSH was measured but results not reported	**Yes?**	Study originally powered for 416 patients by year 2, reported on 218. Menstruation resumption reported but subjectively in an unblinded study. More women attempted pregnancy in the group that received GnRHa but no significant difference in the rate of pregnancy after considering the women who attempted pregnancy
Lymphoma	*Demeestere 2016*	AMH* and Inhibin	**No**	Follow-up 7 years<br>No effect on pregnancies

Only 2 recent studies have reported on pregnancies achieved (Moore et al, 2015 and Demeestere et al. 2016).

The quality of the data on GnRHa has been evaluated in several meta-analyses. In a meta-analysis of available studies up to 2009, only six randomized studies met the inclusion criteria and could be analyzed (Bedaiwy et al, 2011). In that meta-analysis no protective effect of GnRHa was found. In the most recent meta-analysis (Lambertini et al, 2018), which was restricted to women with breast cancer, true individual subject data for amenorrhea were analyzed. Using the data representing 87.1% of the subjects, amenorrhea rates were similar in the GnRHa and the control groups at year one. However, the authors reported an apparent beneficial effect in menstruation resumption at year 2, when the representation of the data was less than 50%. Because it takes approximately 4-6 months for surviving primordial follicles to grow and produce hormones that result in menstruation, any benefit from GnRHa should be reflected in the 6- to 12-month menstruation assessment (Turan et al, 2019). Another shortcoming of this meta-analysis is that each included study had its own definition of ovarian insufficiency, and the meta-analysis did not standardize for diagnosis criteria. Additionally, nearly all studies have reported menstruation resumption as surrogate of ovarian function. However, because none of the available studies were blinded or placebo controlled, the women who received GnRHa may be more likely to interpret any bleeding as normal menstruation (Rodriguez-Wallberg et al, 2016). In the randomized studies that have estimated preservation of ovarian reserve through biochemical gold-standard markers of ovarian reserve, no beneficial effects of GnRHa co-treatment have been confirmed after treatment with chemotherapy. Nevertheless, the proponents of prophylactic GnRHa therapy claim that the GnRHa might antagonize the ovarian cytotoxicity of chemotherapeutics, through unknown mechanisms. If this were true, GnRHa would likely reduce the overall effectiveness of chemotherapy, which does not seem to be the case (Turan et al., 2019).

Detailed data on the most important outcome parameter for a preserved ovarian function, pregnancies after GnRHa co-treatment during cancer treatment, has only been provided in one previous report of a pilot study at Penn University. The authors found a high rate of abortions and chromosome abnormalities in pregnancies occurring in women who received GnRHa co-treatment, suggesting a potential negative effect of the treatment (Fox et al, 2001). There are only two randomized studies that have reported on pregnancies

achieved in groups of women that received GnRHa co-treatment during chemotherapy (Moore et al, 2015; Demeestere et al, 2016) (Table 2). Methodological problems have been recognized in the randomized clinical trial of breast cancer subjects reported by Moore et al. (Moore et al, 2015), such as reduced power, as the study was powered for 416 subjects but reported on 218, as well as the unblinding of GnRHa, which might partially explain the larger number of women that attempted pregnancy in the group who received the GnRHa injection, as they were informed that it could protect their fertility. When considering the information available on the women who actively attempted pregnancy among the groups, the rate of pregnancy was not significantly different between the GnRHa treated and untreated (22 of 25 subjects who received a GnRHa vs. 12 of 18 who did not; Yates's X2=1.73; P=0.188) (Oktay, Rodriguez-Wallberg and Munster, 2015). The most recent randomized clinical trial of women with lymphoma, which has also reported on a long-term follow-up of 7 years (Demeestere, 2016), did not find any effects on sensitive ovarian markers or in the chance to pregnancy in a well characterized cohort of 129 individuals. Up-to date, this is the most complete of all available studies. Outcome measures included both changes in biochemical ovarian markers over time and clinical outcomes included fertility, pregnancy and live births. This study has also the longest subject follow-up of all reported. The inconsistencies among available randomized clinial trial have been highlighted in the recent expert debate article (Dolmans et al., 2020).

2.3. Study rationale

As none of the available randomized studies has been blinded or placebo controlled, and none has previously included young teenagers, who have the greatest ovarian reserve and thus the highest probability to maintain fertility after cancer treatment, and the potentially largest size effect on changes in ovarian reserve, if maintained, our clinical trial is underpinned by a good medical/scientific rationale. The current data on GnRHa co-treatment of women receiving chemotherapy for treatment of cancer are available only on two specific diseases, breast cancer and lymphoma (Hodgkin lymphoma and non-Hodgkin lymphoma (NHL), summarized in Table 2. In this study we will also include subjects receiving less gonadotoxic treatment, such as the primary treatment of acute leukemia, as well as subjects receiving very high gonadotoxic treatment, such as treatment of sarcoma, both diseases with a high incidence among teenagers. All young women (age 18-42) and teenagers (age 14-17) will receive reproductive counseling and may elect to undergo fertility preservation through cryopreservation methods, according to their clinical situation, which is not an exclusion criterion for participation in this trial. It is not known if fertility preservation affects the follow-up levels of AMH, but the procedures should not affect the baseline levels. The use of hormonal contraceptive methods during follow-up may affect hormonal estimations of Estradiol, FSH, LH, but an effect on AMH is unlikely. Both fertility preservation, use of estrogen inhibitors and use/change of contraceptives will be registered in the electronic Case Report Form (eCRF).

The human female ovarian reserve is finite. It is established during gestational life and it decreases continuously until exhaustion, which determines the onset of menopause. Studies of the human female ovarian reserve have recently succeeded in the development of mathematic models that illustrate its reduction from birth to menopause.

In Figure 1, the numbers of follicles in the ovarian reserve pool evidenced on histology at any given age are presented (Wallace et al, 2010). In Figure 2, a validated model of Serum AMH from conception to menopause is presented (Kelsey et al, 2011). The data indicate that AMH is accurately measured also in adolescent girls.

2.4. Study treatment

The study treatment includes the addition of triptorelin (intramuscular injection) or the placebo (intramuscular injection) in addition to the regular disease-specific cytostatic treatment of the subject. The study treatment does not affect the disease-specific cytostatic treatment or treatment regimen, this should proceed according to best practice. All subjects receiving disease-specific cytostatic treatment are eligible for inclusion in the study unless they receive direct radiation of the gonads. Triptorelin/placebo will be given as a complement during the duration of the disease-specific gonadotoxic treatment. Treatment regimens currently in use and the duration of disease-specific cytostatic treatment for the different diagnoses is summarized in table 3 and 4. The first dose of triptorelin/placebo is given as an intramuscular injection -14 to +7 days from the start of the disease-specific gonadotoxic treatment.

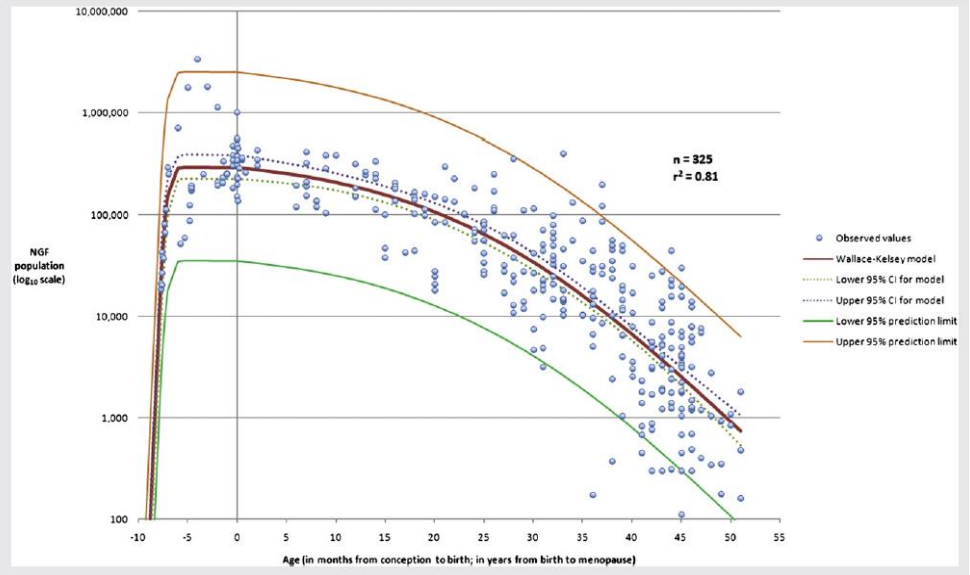

Figure 1. Model of nongrowing follicle (NGF) populations from conception to menopause.
The figure shows the dataset (n = 325), the model, the 95% prediction limits of the model, and the 95% confidence interval (CI) for the model. The horizontal axis denotes age in months up to birth at age zero, and age in years from birth to 51 years. From: Wallace WH and Kelsey TW. Human ovarian reserve from conception to the menopause. PLoS One, 2010

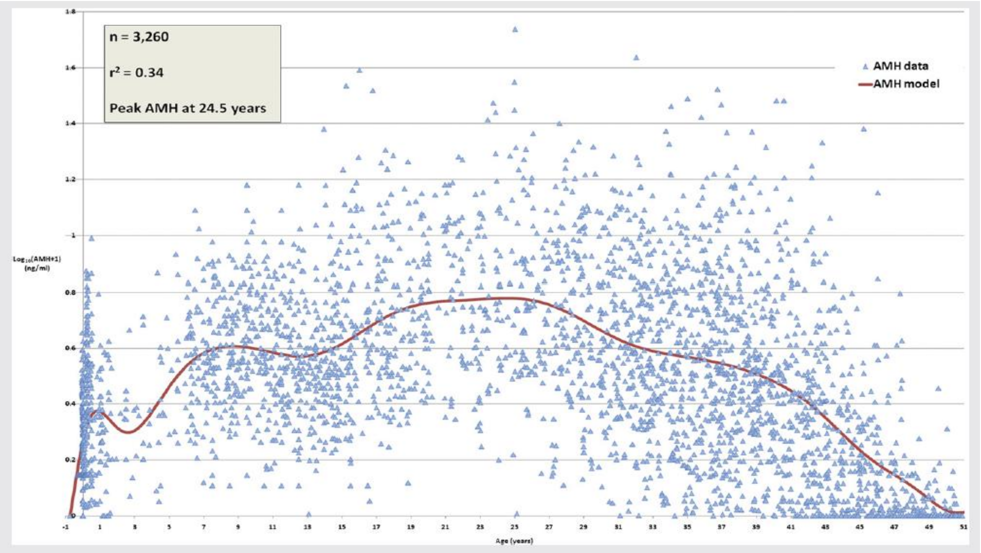

Figure 2. A validated model of serum Anti-Müllerian Hormone (AMH) from conception to menopause. The red line is the model that best fits the 3,260 data points shown as triangles. The coefficient of determination r^2 ¼ 0.34, indicating that 34% of variation in serum AMH concentrations is due to age alone. Peak serum AMH is at 24.5 years. From: Kelsey TW, Wright P, Nelson SM, Anderson RA, Wallace WH. A validated model of serum anti-Mullerian hormone from conception to menopause. PLoS One 2011.

One injection of triptorelin holds a dose that lasts either 3 months (11.25 mg) or 1 month (3.75 mg). The number of doses per subject will be adjusted to cover the disease-specific gonadotoxic treatment with as few injections as possible. The shortest treatment regimen includes disease-specific gonadotoxic treatment for 2 months, which corresponds to 2 triptorelin/placebo injections (2*3.75 mg) and the longest regimen has a disease-specific gonadotoxic treatment for 11 months, which corresponds to 5 triptorelin/placebo injections (2*3.75 mg + 3*11.25 mg). Most study treatments with triptorelin/placebo are between 3-6 months (3-4 injections). The amount of triptorelin/placebo needed is calculated based on the length of each block of disease-specific cytostatic treatment. *Thus, if a disease-specific cytostatic treatment block is four weeks, and the disease-specific gonadotoxic administration of cytostatics is at the 1st day of the block, we will provide a triptorelin/placebo dose high enough to cover the full block to ensure that the disease-specific cytostatic treatment is cleared from the system before the triptorelin/placebo.* The end of disease-specific gonadotoxic treatment is also the starting point for the follow up samples, and the first follow up is done 6 months after the end of the gonadotoxic treatment (EoT).

2.5. Chemotherapy for breast cancer

All breast cancer subjects treated with gonadotoxic chemotherapy will be included in this study. Study treatment will be given from treatment start for the duration of the gonadotoxic treatment blocks. The current chemotherapy regimens for breast cancer include Cyclophosphamide (CPA) which has demonstrated gonadotoxic effects. All subjects receiving CPA will be given triptorelin/placebo from treatment start (-14 to +7 days) until completion of their Fluorouracil, Epirubicin and Cyclophosphamide (FEC), Epirubicin and Cyclophosphamide (EC) or Taxotere and Cyclophosphamide (TC) therapy which lasts between 8-18 weeks depending on the regimen.

Study Code: ProFertil
Version No: 2.5
Date: 2022-09-27
EudraCT No: 2020-004780-71

Table 3. Currently used regimens for treatment of sarcomas, lymphomas and leukemias in children between 14-18 years of age. Suggested start and duration of study treatment (triptorelin/placebo) indicated.
OBS: TS=Treatment Start eg. start of chemo

Diagnosis	**Treatment arm**	**Regimen**	**Gonadotoxic regimen (or cytostatic)**	**Duration of gonadotoxic treatment**	**Accumulated dose (max/m2)**	**Risk of infertility**	**Approx. duration of triptorelin /placebo**	**Start of triptorelin /placebo**
Osteosarcoma	All	Euramos- 1	MAP	21 w	480mg CisP	High	6 mon	TS
Ewing sarcoma	All	Ewing 2008	VIDE + VAC	44 w	18000 mg I 12000 mg C	High	11 mon	TS
Soft tissue sarcoma	Subgoup B, subgroup C (with IR)	CWS guidance	Ifosfamide	13 v	12000mg I	Intermediate	4 mon	TS
Soft tissue sarcoma	Subgroup C (no IR), subgroup D-H, SySa, STET, UDS, NRSTS HR	CWS guidance	Ifosfamide	25v	18000mg I	High	6 mon	TS
Hodgkin lymphoma	TL-2	EuroNet-PHL-C2	CPA	4 mon	2000-2500 mg C	low	4 mon	TS
Hodgkin lymphoma	TL- 3	EuroNet-PHL-C2	CPA	6 mon	4000-5000 mg C	Intermediate	6 mon	TS
Lymfoblast lymphoma	Standard risk group I/II	LBL2018	CPA	2 mon	2000 mg C	Low	4 mon	TS
Lymfoblast lymphoma	Standard risk group, High risk group (standard arm)	LBL2018	CPA	5 mon	3000 mg C	Low	7 mon	TS
Lymphoblast lymphoma	High risk group (intensified arm)	LBL2018	CPA, Ifosfamide	5 mon	4000 mg C, 4000 mg I	Intermediate	7 mon	TS
B-cell lymphoma	R1	B-NHL 2013	CPA, Ifosfamide	2,5 mon	1000 mg C, 4000mg I	Low	3 mon	TS
B-cell lymphoma	R2-I/II, R2-III	B-NHL 2013	CPA, Ifosfamide	4,5 mon	2000 mg C 8000 mg I	Intermediate	6 mon	TS
B-cell lymphoma	R3	B-NHL 2013	CPA, Ifosfamide	6 mon	2000 mg C 8000 mg I	Intermediate	6 mon	TS
Anaplastic Large Cell Lymphoma	All	ALCL-99	CPA, Ifosfamide	6 mon	3400 mg C 12000 mg I	Intermediate	6 mon	TS
AML	Eligable for transplant	NOPHO-DBH AML-2012	SCT	-	TBI (C)	High	3 mon	At indication for SCT
ALL	HR Eligable for transplant	ALLTogether	SCT	-	TBI (C)	High	3 mon	At indication for SCT
ALL	VLR, IR	ALLTogether		6 mon		Low	6 mon	TS

Study Code: ProFertil
Version No: 2.5
Date: 2022-09-27
EudraCT No: 2020-004780-71

Table 4.Currently used regimens for treatment of sarcomas, lymphomas and leukemias in adults between 18-39 years of age. Suggested start and duration of study treatment (triptorelin/placebo) indicated.
OBS: TS=Treatment Start eg start of chemo

Diagnosis	**Treatment arm**	**Regimens**	**Treatment regimen(gonadotoxic)**	**Duration of gonadotoxic treatment**	**Accumulated max dose/m²**	**Risk of infertility**	**Duration of triptorelin /placebo**	**Start of triptorelin /placebo**
Osteosarcoma	High grade	Euramos 1	MAP	21 w	480mg CisP	High	6 mon	TS
Ewing sarcoma	Cyt. sensitive tumors	Ewing 2008	VIDE + VAC	44 w	18000 mg I<br>12000 mg C	High	11 mon	TS
Ewing sarcoma	Cyt. sensitive tumors	EuroEwing 2012	VDC/IE	28w	8400 mg C<br>63000 mg I	High	7 mon	TS
Soft tissue sarcoma	Subgoup B, subgroup C (with IR)	CWS guidance	Ifosfamide	13w	12000 mg I	Intermediate	4 mon	TS
Soft tissue sarcoma	Subgroup C (no IR), subgroup D-H, SySa, STET, UDS, NRSTS HR	CWS guidance	Ifosfamide	25w	18000 mg I	High	6 mon	TS
Hodgkin lymphoma	IA, IIA, IB	ABVD*2	ABVD	8-16 w	-	Low	3 mon	TS
Hodgkin lymphoma	IIB–IV	BEACOPP escalated	BEACOPP escalated	13-19w	5000-7500 mg C	Intermediate	4-6 mpn	TS
Hodgkin lymphoma	IIB–IV	Alternative BEACOPP	AVBD/AVD AVBD/BEACOPP	20-24w	3900-5000 mg C	Intermediate	5-6 mon	TS
Lymphoblast lymphoma	All risk groups	NOPHO-08	Stratification and treatment arms according to NOPHO		At high risk, TBI (C)	Low/ Intermediate /high	3-9 mon	TS
B-cell lymphoma	aaIPI=0		R-CHOPx4 or R-CHOP-21x6 or R-CHOP-21x4	12-18 w	3000-4500 mg C	Intermediate	3-5 mon	TS
B-cell lymphoma	aaIPI=1		R-CHOP-14x6 or R-CHOP-21 x6	12-18 w	4500 mg C	Intermediate	3-5 mon	TS
B-cell lymphoma	aaIPI=2<br>aaIPI=3		R-CHOEP-14x6 or R-CHOP/MTX-21x2 + RCHOEP-14x4, R-HyperCVAD	12-14 w	4500 mg C	Intermediate	3-4 mon	TS
AML	Indication for SCT	DA3+5, AIDA	SCT	-	TBI (C)	High	3 mon	At indication for SCT
ALL	HR-hSTC	ALLTogether	SCT	-	TBI (C)	High	3 mon	At indication for SCT
ALL	VLR. IR	ALLTogether		6 mon		Low	6 mon	TS
Breast cancer	CYT HER 2-	Adjuvant and neoadjuvant theraphy	$E_{75}C$, $T_{75}C$, $_{dd}EC$, $_{d}E_{90}C$, $E_{100}C3$,	8-18 w	1500-3600 mg C	Intermediate	3-5 mon	EC, FEC, TC
Breast cancer	CYT HER2+	Adjuvant and neoadjuvant theraphy	$E_{100}C3$, $_{d}E_{90}C$, $_{dd}EC$,	8-12 w	1500-2400 mg C	Intermediate	2-3 mon	EC, FEC, TC

2.6. Chemotherapy for acute leukemias

Subjects planned for treatment for Acute Myeloid Leukemia (AML) and Acute Lymphatic Leukemia (ALL) will be included in this study. For subjects with ALL stratified of Very Low Risk or Intermediate risk according to their chemotherapy treatment, the study treatment will be given from chemotherapy start and will be continued for six months. The subjects with AML with indication for stem cell transplantation (SCT) as well as ALL subjects planned for High Risk chemotherapy will receive the study treatment from the time SCT is indicated and until the end of the intense gonadotoxic treatment (approximately 3 months with current regimens).

Rodriguez-Wallberg KA, *et al. BMJ Open* 2023; 13:e078023. doi: 10.1136/bmjopen-2023-078023

ALL is currently treated according to the ALLTogether regimen and subjects with AML with NOPHO-Dutch-Belgien-Holland (DBH)-AML-2012 or ALL-Trans Retinoic Acid and Idarubicin (AIDA) or Daunorubicin and Cytarabin (DA 3+5). For both ALL and AML, all subjects assigned SCT will be eligible for inclusion. The decision to proceed with SCT is based on several criteria, but risk group and age are main factors to support the decision. Approximately 50% of the subjects with AML receive SCT as well as most subjects from the High Risk (HR) group of ALL. Triptorelin/placebo treatment should start before the conditioning before allogeneic SCT as this is highly gonadotoxic, both through chemotherapy (CPA) and Total Body Irradiation (TBI). Most subjects, regardless of age, experience amenorrhea after the conditioning treatment.

Subjects with ALL from the Very Low risk (VLR), Standard Risk (SR) and Intermediate Risk (IR) groups (ALLTogether) will also be included. They rarely get SCT and only receive a mildly gonadotoxic treatment. Triptorelin/placebo will be given from cytostatic treatment start (-14 to +7 days) and until the start of Maintenance phase (Approximately 6 months).

2.7. Chemotherapy for lymphomas

There are a wide range of lymphomas, where Hodgkin lymphoma is the most common among young individuals. All subjects with lymphoma treated with chemotherapy regimens based on CPA, Ifosfamide or a combination of both can be included in this study. Also subjects receiving SCT are eligeable for inclusion. Study treatment will be given from treatment start for the duration of the gonadotoxic treatment blocks. Most treatment regimens have a low to intermediate effect on fertility. *Chronic lymphomas and subjects with direct radiation to the gonads will be excluded from the study.*

The current regimen for treating Hodgkin lymphoma in children is European Network Pediatric Hodgkin Lymphoma (EuroNetPHL)-2012. Treatment arm TL-2 and TL-3 will be included as they receive CPA during 4-6 months. Triptorelin/placebo will be given from start of treatment.

Adults with Hodgkin lymphoma are currently treated with Adriamycin (Doxorubicin), Bleomycin, Vinblastine, Dacarbazine (ABVD) or Bleomycin, Etoposide, Adriamycin (Doxorubicin), Cyclophosphamide, Oncovin (Vincristine), Procarbazine, Prednisone (BEACOPP), from those regimens risk group IA, IIA, IB, IIB-IV will be included. They will receive triptorelin/placebo for 3-6 months depending on regimen.

B-cell lymphoma in children is currently treated according to the B-cell Non-Hodgkin Lymphoma (B-NHL)-2013 regimen, and all treatment arms will be included. B-NHL-2013 includes a combination of CPA and Ifosfamide. Triptorelin/placebo will be given from treatment start and 3-6 months onward depending on the risk group.

B-cell lymphoma in adults is treated with Cyclophosphamide (CPA), Adriamycin (Doxorubicin), Oncovin (Vincristine), Prednisone (CHOP) or Cyclophosphamide, Adriamycin (Doxorubicin), Oncovin (Vincristine), Etoposide, Prednisone (CHOEP), both CPA based regimens as well as with R-HyperCVAD, rituximab, cyklofosphamide and vinchristine, doxorubicin, dexametason and methotrexate combined with R-Metotrexate/HIDAC (rituximab, metotrexate cytarabine). Triptorelin/placebo will be given from treatment start and 3-5 months onwards.

Lymphoblastic lymphoma (LBL) is commonly treated according to LBL2018 in children and NOPHO -08 in adults; all risk groups are included. Treatment is given usually with CPA or CPA and Ifosfamide (in the HR Intensified arm). Treatment lasts from 2-5 months and all subjects should be included at treatment start of LBL2018.

Anaplastic large cell lymphoma (ALCL) is currently treated according to ALCL-99. All groups are included and treatment with CPA and Ifosfamide lasts for 6 months. Triptorelin/placebo is given from treatment start (-14 to +7 days).

2.8. Chemotherapy for sarcomas

Sarcomas are generally treated with a high dose gonadotoxic cytostatic treatment, that gives a correspondingly high risk for infertility. Ifosfamide in high doses is the most common treatment but also Cisplatin and CPA are used in some regimens. Study treatment will be given from treatment start for the duration of the gonadotoxic treatment blocks. Subjects with direct radiation of the gonads will be excluded from this study.

Osteosarcoma is currently treated according to European and American Osteosarcoma Study (Euramos 1). Subjects with high grade osteosarcoma are included as they are given Cisplatin for 21 weeks. Triptorelin/placebo will be given from treatment start and 6 months onwards.

Ewings sarcoma is currently treated either according to Ewing 2008 or Euro Ewing 2012. Both regimen give a combination of CPA and Ifosfamide but with different intervals and different dosage. All subjects treated with cytostatic treatment will be included. Treatment according to Ewing 2008 lasts for 44 weeks and triptorelin/placebo will be given from treatment start and 11 months onwards. Treatment according to Euro Ewing 2012 lasts for 28 weeks and triptorelin/placebo will be given from treatment start and 7 months onwards.

Soft tissue sarcoma is currently treated according to Cooperative Weichteilsarkom Studiengruppe (CWS) guidance and all subgroups except A are included in this study. Subjects are treated with Ifosfamide between 13-25 weeks and will be given triptorelin/placebo from treatment start (-14 to +7 days) and 4-6 months onwards.

3. Risk-benefit evaluation

3.1. Risks

Usage of GnRHa has shown to give a higher incidence of hot flashes and sweating in adult subjects. As both cytostatic treatment and GnRHa may affect the hormonal levels, the onset of menopausal symptoms may be faster and possibly stronger when adding triptorelin in paralell with the cytostatic treatment. Differences between these symptoms among the groups will be investigated. Triptorelin might also affect bone density, especially if used for a long-term period. Thus, we have chosen to exclude subjects with osteoporosis and subjects previously or currently diagnosed with anorexia. We will also document changes in bone mineral density to evaluate if the short term usage proposed in this study would have a measurable negative effect in bone mass. Other known side effects of GnRHa are reduced libido, vaginal bleeding, headache and hyperhidrosis. Anaphylactic reactions are rare and any effects of overdoses not documented. 5% of the users experience swelling, edema, redness and soreness at the site of injection. All subjects will be given the 1-month injection as 1st time.

3.2. Benefits

Young cancer survivors have ranked infertility after cancer treatment as one of the most difficult late effects of their treatments. The subjects in this study will receive fertility preservation counseling, according to current Swedish guidelines (Rodriguez-Wallberg et al, 2019b) and may elect to undergo fertility preservation methods within the framework of the standardized health care services. Individualized counselling will be provided during

follow-up. Identification of individual predictors of ovarian toxicity and infertility after cancer treatment identified in this study may be applied in the future for subject selection towards the need of fertility preservation interventions, hence reducing potential risks for subjects who do not require those treatments and reducing costs in health care.

The participants in this study will also have the opportunity to receive a potentially effective fertility protective treatment, which otherwise is not given as a standard routine. The results of published articles are still contradictory. The subjects will be carefully informed that there is no proof of a beneficial effect of GnRHa on female fertility, nor any proof on the contrary either.

3.3. Risk-Benefit Assessment

As the benefit of GnRHa is still in dispute, solving this dispute is the sole purpose of this study. The subjects will also get access to established methods for fertility preservation and counselling. In this context, the subjects are not going to miss a validated option and will also have the possibility to gain a benefit if the GnRHa triptorelin could decrease the risk of future Premature Ovarian Insufficiency (POI) and protect their future fertility. In a recent meta-analysis, GnRHa co-treatment given during chemotherapy was judged as not dangerous (Lambertini et al, 2018), nevertheless the GnRHa preparations resulted in adverse effects, such as sweating and hot flashes, that impaired cancer subjects' quality of life. For this reason we will also include symptoms records during chemotherapy treatment and follow-up. The study treatment is administered as an intramuscular injection, which could cause soreness at the injection site. Most of the tests in this study are non-invasive, but we are including extra blood samples. This might be seen as both an inconvenience but also an opportunity to get extra data and monitor individual fertility. Thus, the overall risk for the individuals due to participating in the study is low but possibly the benefit as well, especially if the subject has no reproductive wish.

The main benefits of the study are on a larger scale as we hope to be able to either establish or refute GnRHa as a viable method for fertility preservation and managing this goal will have effects on future fertility treatment regimes both nationally and globally. Thus, on a whole we conclude that the overall benefits of the study surpasses the fairly low risk for the study subjects.

4. Study objectives

The question that we wish to investigate is an important question born from our clinical practice and of high relevance for young women (age 18-42) and teenagers (age 14-17) with cancer. Among all sequelae of cancer treatment, infertility is a recognized issue with high negative impact in the quality of life cancer of cancer survivors. If GnRHa treatment can be associated with maintained fertility, such treatment would be particularly suitable for the very young subjects or for subjects that require immediate cancer therapy and do not have the opportunity to perform any established and time-demanding fertility preservation procedures. However, if the GnRHa treatment doesn´t show any protective effects, research should focus on finding therapies that can prevent infertility in our youngest cancer subject population.

Why has it been such a tremendous difficulty assessing the effect of GnRHa? The main reason is the lack of studies of high quality with an appropriate experimental design and pre-defined reproductive outcomes. These should include estimates of the preservation of ovarian follicles after chemotherapy, and also fertility (pregnancy and live birth), as main outcomes. An

important issue is that the few randomized trials currently available have all been unblinded. Hence, all women that have received GnRHa co-treatment in randomized studies have been informed about the aim to preserve their fertility through that treatment.

A placebo-controlled trial is thus highly needed, and it would provide the solid knowledge that is currently lacking. Importantly, all available studies have included adult women up to the end of their reproductive age span, which raises the question about the dubious preservation of fertility in women above 40-years of age receiving chemotherapy treatment. Due to the natural high ovarian reserve in women of younger age and its natural depletion with increasing age, with exhaustion of the follicle pool determining the onset of menopause, inclusion of younger women and teenagers in this randomized study, would enhance the possibility to identify a significant preservation of the remaining follicle pool after a chemotherapy insult.

Modern biochemical markers of ovarian reserve, currently considered gold standard, will be used for this estimation. The study designed is much improved, in contrast with the available studies, as it would be multicentre, randomized, placebo-controlled and double-blinded. Appropriate fertility endpoints and outcomes in this cohort could be prospectively followed-up over the years, including oncologic outcomes and co-morbidities.

The debate on ovarian protection using GnRHa has gone on for over 35 years and our purpose is to investigate if the administration of GnRHa during cancer treatment can preserve the fertility of young female cancer subjects.

4.1. Primary objective

To estimate the changes in ovarian reserve following chemotherapy for treatment of cancer with or without GnRHa by determination of the AMH at 12 months after end of gonadotoxic treatment (EoT) in women with breast cancer.

4.2. Secondary objectives

Key secondary objective:

To estimate the changes in ovarian reserve following chemotherapy for treatment of cancer with or without GnRHa by determination of the AMH at 12 months after EoT in women with acute leukemias, lymphomas and sarcomas.

Secondary objectives (to be evaluated at EoT, 6, 12 months after EoT and continuously during follow-up years 2, 3, 4, 5 after EoT):

- To estimate the changes in ovarian reserve with or without GnRHa by determination of the antral follicle counts (AFC) in women with breast cancer
- To estimate the changes in ovarian reserve with or without GnRHa by determination of the AFC in women with acute leukemias, lymphomas and sarcomas
- To estimate the changes in ovarian reserve with or without GnRHa by longitudinal observation of AMH levels in women with breast cancer

- To estimate the changes in ovarian reserve with or without GnRHa by longitudinal observation of AMH levels in women with acute leukemias, lymphomas and sarcomas

- To compare the proportion of females with or without GnRHa that develop ovarian insufficiency by determination of FSH, inhibin and estradiol at standardized timepoints

- To investigate the impact of BMI, use of contraceptives and endocrine adjuvant therapy in changes of ovarian reserve with or without GnRHa by longitudinal observation of AMH levels, FSH, inhibin and estradiol at standardized timepoints

- To investigate the effect of GnRHa with or without GnRHa on ovarian blood supply

- To estimate the proportion of females with or without GnRHa that develop amenorrhea (no menstruations)

- To investigate fertility and childbirth after cancer treatment in women with or without GnRHa who attempt pregnancy during follow-up

- To determine health-related QoL with or without GnRHa during chemotherapy and after cancer treatment

  To study development of co-morbidities during follow-up and bone mineral density after completion of cancer treatment with or without GnRHa

- To evaluate disease-specific oncologic outcomes, recurrence rate, overall survival and disease-free survival with or without GnRHa

4.3. Primary endpoint

The difference in recovery of AMH levels at follow-up 12 months after EoT, relative to AMH levels at EoT, between the GnRHa group and the placebo group in women with breast cancer.

4.4. Secondary endpoints

Key secondary endpoint:

The difference in recovery of AMH levels at follow-up 12 months after EoT, relative to AMH levels at EoT, between the GnRHa group and the placebo group in women with acute leukemias, lymphomas and sarcomas.
Secondary endpoints:

- Comparison of AFC measured by ultrasound at EoT, 6, 12 months after EoT and continuously during follow-up years 2, 3, 4, 5 after EoT, between the GnRHa group and the placebo group:
    - In women with breast cancer
    - In women with acute leukemias, lymphomas and sarcomas

- The difference in recovery of AMH levels at 6 months, and follow-up years 2, 3, 4, 5 after EoT, between the GnRHa group and the placebo group:
    - In women with breast cancer
    - In women with acute leukemias, lymphomas and sarcomas

- Comparison of FSH, inhibin and estradiol performed at EoT, 6, 12 months after EoT and continuously during follow-up years 2, 3, 4, 5 after EoT, between the GnRHa group and the placebo group:
    - In women with breast cancer
    - In women with acute leukemias, lymphomas and sarcomas

- Comparison of blood flow to the ovarian artery (right and left Doppler flow PI, RI) at baseline, EoT, 6, 12 months after EoT and continuously during follow-up years 2, 3, 4, 5 after EoT, between the GnRHa group and the placebo group:
    - In women with breast cancer
    - In women with acute leukemias, lymphomas and sarcomas

- Comparison of the proportion that develop amenorrhea (no menstruations) at EoT, 6, 12 months after EoT and continuously during follow-up years 2, 3, 4, 5 after EoT, between the GnRHa group and the placebo group:
    - In women with breast cancer
    - In women with acute leukemias, lymphomas and sarcomas

- Investigation of the impact of BMI, use of contraceptives and endocrine adjuvant therapy in changes of ovarian reserve with or without GnRHa by longitudinal observation of AMH levels, FSH, inhibin and estradiol at standardized timepoints

- Comparison of of pregnancy wish, pregnancy attempts and pregnancy outcomes at EoT, 6, 12 months after EoT and continuously during follow-up years 2, 3, 4, 5 after EoT, between the GnRHa group and the placebo group:
    - In women with breast cancer
    - In women with acute leukemias, lymphomas and sarcomas

- Comparison of health-related QoL, sexuality and reproductive health examined at EoT, 6, 12 months after EoT and continuously during follow-up years 2, 3, 4, 5 after EoT, between the GnRHa group and the placebo group:
    - In women with breast cancer
    - In women with acute leukemias, lymphomas and sarcomas

- Comparison of bone mineral density at baseline, EoT and 12 months after EoT and follow-up year 5, between the GnRHa group and the placebo group:
    - In women with breast cancer
    - In women with acute leukemias, lymphomas and sarcomas

- Investigation of recurrence rate, overall survival and disease-free survival at 12 months after EoT and follow-up years 2, 3, 4, 5 after EoT, between the GnRHa group and the placebo group:
  - In women with breast cancer
  - In women with acute leukemias, lymphomas and sarcomas

5. Study design and procedures

5.1. Overall study design

This study is a randomized, double-blinded, placebo-controlled, phase III study, which will also include young teenagers from 14 years of age that have had completed pubertal development including menarche. See section 7.5 for more information on the procedure for randomization. The study design is shown in the study flow chart (Table 5) and in the study scheme (Figure 3). The study investigates the protective effect of GNRHa (Study drug triptorelin) from cytotoxic damage on the ovarian reserve as primarily represented by the recovery of AMH levels after completed gonadotoxic cytostatic treatment (EoT). Recovery of AMH will be compared with a parallel randomized control group receiving placebo. See section 7, study treatments for more detailed information. Study treatment will proceed for 2-11 months depending on diagnosis. The two study arms will be treated for their cancer according to best practice in parallel with study treatment or placebo. Each subject will be followed for 12 months after EoT to achieve the primary endpoint, and the total study time is 5 years from EoT. A primary analysis of all endpoints will be done after completion of the primary endpoint and a final analyses after end of the 5 year follow up.

Figure 3 Study scheme

Visit	1	2	Treatment visits	3	4, 5	6, 7, 8, 9
	**Screening/ Baseline**	R	**Treatment A: Triptorelin (n=250)**	**End of Treatment**	**Follow up**	**Follow up**
			**Treatment B: Placebo (n=250)**	**End of Treatment**		
**Time:**	**<-1 month**	**0**	**Duration: 2-11 months**	**2-11 months after initiation of gonadotoxic treatment**	**6, 12 months after EoT**	**2, 3, 4, 5 years after EoT**
	Screening/ inclusion	Randomization and start of study treatment and gonadotoxic treatment	Study treatment		Evaluation	Evaluation

5.2. Procedures and flow chart

5.2.1. Assessments and procedures

Informed consent

At the screening/baseline visit the subject will be provided with written and oral information about the study, including objectives, procedures and possible risks and benefits of study participation. If the subject decides on participation, the informed consent form is signed by the subject and the principal investigator or sub-investigator before any study-related activities are carried out.

Fertility preservation

All young women (age 18-42) and teenagers (age 14-17) will receive reproductive counseling and may elect to undergo fertility preservation through cryopreservation methods, according to clinical recommendations. This is not an exclusion criterion for participation in this study.

Demography, vital signs, ECG and bone mineral density measurement

Demographic data such as age, weight and height will be collected at screening. Inspection of injection site and measuring of blood pressure and heart rate will be performed at the screening/baseline visit and at every study visit throughout the study. At screening/baseline an ECG will be performed with follow-up at EoT. At screening/baseline a bone mineral density measurement will be performed with follow-up at EoT, and 12 months and 5 years after EoT. All data will be collected and noted in the medical record by the examining physician/nurse.

Pregnancy test

At screening/baseline all subjects must perform a pregnancy test (blood sample) to exclude pregnancy before they are enrolled in the study.

Medical history

A complete medical history will be recorded at the screening/baseline visit. The medical history will include a review of all past and current relevant diseases and diagnoses.

Safety blood samples

Blood samples for measurement of routine blood parameters (a complete blood count, including B-hemoglobulin, WBC, differential count and platelets), liver status (ALT, AST, bilirubin, ALP, GGT), renal status (Sodium, Potassium, Calcium, Creatinine, Albumin) will be collected at the screening/baseline visit, at EoT and as part of clinical routine during chemotherapy.

Research samples (biochemical markers)

Measurements of different hormonal markers will be performed, such as AMH, FSH, Inhibin, Estradiol and LH at screening/baseline visit and at EoT and continuously at follow up 6, 12 months, and follow-up years 2, 3, 4 and 5 after EoT. Samples will be taken during the first days of the menstrual cycle in women with spontaneous menstruation. Women with amenhorrea can provide samples at any time point. Subjects treated with contraceptives should if possible quit contraceptives 8 weeks before blood sampling. Continous use of contraceptives is not an exclusion critera, but use and changes in use should be noted in the eCRF (See Concomitant medication).

Ultrasound

To count the antral follicles and measure the ovarian blood flow, transvaginal ultrasound will be performed at screening/baseline visit and at visits at EoT, at follow up 6, 12 months after EoT and continuously at follow up years 2, 3, 4 and 5 after EoT.

Questionaire including health-related quality of life

To collect reproductive outcome data, such as reproductive health, sexuality and quality of health aspects, pregnancy wish, pregnancy attempts and pregnancy outcomes, a specifically developed validated questionnaire and checklist including EORTC QLQ C30, Hospital Anxiety and Depression Scale (HAD) and Female Sexual Function Index (FSFI) (only for sexually active subjects and those above 18) will be completed at screening/baseline visit and follow up visits at EoT, at follow up 6, 12 months after EoT and years 2, 3, 4 and 5 after EoT. EORTC QLQ C30: The EORTC quality of life questionnaire (QLQ) is an integrated system for assessing the health-related QoL (HRQoL) of cancer subjects participating in international clinical studies.

Concomitant medication

Information regarding prior and concomitant medications (outside of treatment protocol, study protocol and diagnosis-specific cancer protocol) including ongoing fertility preservation measures, use of contraceptives and hormonal treatments will be collected at screening/baseline visit. During follow up, data will be collected on use of contraceptives and hormonal treatments including adjuvant endocrine treatment for breast cancer (tamoxifen, aromatase inhibitors). The investigator or designee will assess changes in concomitant medications throughout the study by asking the subject at each visit. Any changes reported by the subject will be recorded in the eCRF.

5.2.2. Study visits

Visit 1 – Screening/baseline

A screening/baseline visit will take place not more than one month before study enrollment. Before any study related procedures are performed the subject will sign the informed consent form. The investigator will evaluate all eligability criteria for enrollment. All subjects will perform a pregnancy test, to exclude pregnancy. All subjects will receive reproductive counseling and they may elect to undergo fertility preservation through cryopreservation methods, according to their clinical situation. This is not an exclusion criterion for enrollment. Subsequently, a physical examination, ECG and transvaginal ultrasound for measurement of AFC and measurement of ovarian blood flow will be performed, demographic data will be collected and vital signs measured. A bone mineral density measurement will be scheduled to be performed as soon as possible. Medical history and prior/concomitant medications will be reviewed. Blood samples for safety and biochemical markers for research will be collected. All subjects will be asked to complete the HRQoL baseline validated questionnaire.

Visit 2 – Day 0 (-14 to +7 days from start of gonadotoxic treatment), randomization and start of study treatment and gonadotoxic treatment (or consolidation phase for subjects with HR ALL and AML)

If all inclusion criteria and no exclusion criteria are met, subjects will be enrolled and randomized according to the study setting. Subjects randomized to treatment A will receive intramuscular injection(s) of triptorelin, 3.75 mg/month or 11.25 mg/3 months during the duration of the gonadotoxic treatment. Subjects randomized to treatment B will receive intramuscular injection(s) of placebo (NaCl 0.9%) during the duration of the gonadotoxic treatment. All subjects will initially receive a one-month injection (triptorelin, 3.75 mg/month or NaCl 0,9%).

All subjects will start receiveing disease-specific gonadotoxic chemotherapy between one week before and up until two weeks after first study treatment, either chemotherapy for breast cancer or acute leukemias (ALL or AML either at time of primary treatment or relapse), lymphomas (Hodgkin and non-Hodgkin) or sarcomas (osteo and Ewing). The subjects will be assigned a risk group by the treating physician (Low, Intermediate or High risk for infertility) with regard to the expected effect on fertility (based on cumulative dose cytostatics and irradiation; see examples in Tables 3 and 4). Vital signs and the latest safety blood samples will be checked, concomitant medications will be reviewed and any adverse events such as allergic reactions to the study drug will be registered in the eCRF.

Treatment Visits – 0-4 visits every 1-3 months (±7 days) until end of gonadotoxic treatment block

Subjects randomized to treatment A will receive intramuscular injection(s) of triptorelin, 3.75 mg/month or 11.25 mg/3 months during the duration of the gonadotoxic treatment. (This may include 0-4 visits depending on the duration of the gonadotoxic treatment). Subjects randomized to treatment B will receive intramuscular injection(s) of placebo (NaCl 0.9%) during the duration of the gonadotoxic treatment. Vital signs and the latest safety blood samples will be checked, concomitant medications will be reviewed and adverse events as well as any allergic reactions to the study drug registered in the eCRF.

Visit 3 – End of gonadotoxic treatment at 2-11 Months (±1 month after finalized cycle of gonadotoxic treatment and study treatment)

At end of study treatment with either triptorelin or placebo, coinciding with the end of disease-specific gonadotoxic treatment, the following procedures are carried out: ultrasound, bone mineral density, ECG, vital signs, safety blood samples are collected, and biochemical markers are measured. A validated questionnaire including data on reproductive health, sexuality and quality of health aspects, pregnancy wish, pregnancy attempts and pregnancy outcomes (childbirth) is completed, concomitant medications and adverse events are reviewed and registered.

Visit 4-9 – Follow up: 6, 12 months and 2, 3, 4, 5 years (±1 month) after EoT

The following procedures are carried out during each follow up visit: Vital signs, ultrasound, and biochemical markers measured through research samples. HRQoL follow up validated questionnaire is completed, including data on reproductive health, sexuality and quality of health aspects, pregnancy wish, pregnancy attempts and pregnancy outcomes (childbirth). Concomitant medications and adverse events are reviewed. Oncologic outcomes are included in the eCRF, as well as overall survival, relapse-free survival and co-morbidities. Bone mineral density is measured at 12 months after EoT and at follow up year 5.

Study Code: ProFertil
Version No: 2.5
Date: 2022-09-27
EudraCT No: 2020-004780-71

Table 5 Flow chart of study visits and study procedures

Procedure	**Visit 1** *Screening Baseline*	**Visit 2** *Day 0 (-14/±7 days) Initiation of study treatment (triptorelin/ placebo)/ gonadotoxic treatment*	**Treatment visits** *During gonadotoxic treatment (triptorelin /placebo) (0-4 additional visits for study treatment) (±7 days)*	**Visit 3** *EoT (±1 month) End of gonadotoxic treatment (1 or 3 months after last admininstrati on of triptorelin/ placebo)*	**Visit 4** *6 Months (±1 months) From EoT*	**Visit 5** *12 Months (±1 months) From EoT*	**Visit 6** *24 Months (±1 months) From EoT*	**Visit 7,8,9** *3,4,5 Years (±1 months) From EoT*
Inclusion/ exclusion criteria	X	X						
Informed consent	X							
Fertility preservation	X							
Demography	X							
Physical examination	X							
Vital signs	X	X	X	X	X	X	X	X
Medical history	X							
Randomization		X						
Safety blood samples¥	X			X				
Pregnancy test	X							
Biochemical markers€	X			X	X	X	X	X
Bone mineral density	X			X		X		X#
Ultrasound£	X			X	X	X	X	X
ECG	X			X				
Study treatment*		X	X					
Questionaire (Baseline)	X							
Questionaire (Follow up)				X	X	X	X	X
Concomitant medication	X	X	X	X	X	X	X	X
Adverse Events (AE & SAE)		X	X	X	X	X	X	X

* *Subjects randomized to treatment GnRHa will receive intramuscular injection(s) of triptorelin, 3.75 mg/month or 11.25 mg/3 months during the duration of the gonadotoxic chemotherapy, subjects randomized to placebo will receive intramuscular injection(s) of placebo (NaCl 0.9%).* £ *Antral Follicle Counts and Ovarian Doppler Flow*
# *Only at year 5 of follow up*
¥ *Safety blood samples are taken continuously during the cancer treatment and data is recorded in the eCRF. However, only safety blood samples collected at baseline and end of treatment are included in the study.*
€ *Research samples including AMH, Follicle Stimulating Hormone (FSH), Inhibin, Estradiol and LH*

5.3. Biological sampling procedures

5.3.1. Handling, storage, and destruction of biological samples

Research samples (8 x 20 ml) for measurement of AMH, FSH, inhibin and estradiol will be collected, frozen and stored at each site before batch analyses at Karolinska Institutet/Karolinska University Hospital. AMH will be analyzed with the fully automated AMH assay developed on the Elecsys/Cobas electrochemiluminescence immunoassay platform available at Karolinska Institutet or Karolinska University Hospital. FSH, inhibin and estradiol will be estimated by ultra-sensitive assay at Karolinska Institutet or Karolinska University Hospital.

5.3.2. Total volume of blood per study subject

The total volume of blood taken from each subject during the study is 200 ml. 40 ml (safety blood samples 2 x 20 ml) and 160 ml (research samples 8 x 20 ml) over a period of 5 years plus the time from Inclusion to EoT (study treatment).

5.3.3. Biobank

All research samples taken for storage in this study are registered in a biobank at Stockholms medicinska biobank at Karolinska University Hospital and handled according to the current biobank laws and regulations. The research samples are coded/pseudonymized to protect the study subject's identification. All research samples and the identification/code list are stored securely and separately to prevent unauthorized persons from having access to them.

5.4. End of Study

The end of study is defined as the last study subject's last follow-up visit. The principal investigator has the right to terminate the study for clinical or administrative reasons at any time. The study may be prematurely terminated due to a high number of serious adverse events (related or not related to the study medication) or if the enrollment process cannot be completed within a reasonable time frame.

Decision on premature study termination or suspension will be made by the sponsor. Study termination will be reported to the MPA within 90 days, or within 15 days if the study is terminated prematurely. The investigators will inform subjects and ensure that the appropriate follow-up is arranged for all involved. A summary report of the study will be submitted to the MPA, within 6 months (due to study involving teenagers) after study termination.

6. Subject selection

6.1. Inclusion criteria

To be included in the study, subjects must meet the following criteria:

- Signed informed consent
- Age 14-42 years at cancer diagnosis

- Female subjects with breast cancer or acute leukemias, lymphomas (Hodgkin and non-Hodgkin) or sarcomas (osteo, soft tissue and Ewing) confirmed by histology and assigned for diseace-specific chemotheraphy
- Confirmed menarche
- ECOG performance status 0-1
- Adequate bone marrow, renal, hepatic and cardiac functions and absence of other uncontrolled medical or psychiatric disorders

6.2. Exclusion criteria

Subjects must not be included in the study if any of the following criteria are met:

- Ongoing treatment with GnRHa at baseline
- Demonstrated premature ovarian failure at time of randomization according to clinical or biochemical data
- Previous or planned bilateral oophorectomy
- Pregnancy or breastfeeding at time of start of chemotherapy
- Other malignancy diagnosed within the last five years
- Uncontrolled hypertension, heart, liver, kidney related or other uncontrolled medical or psychiatric disorders including previous or current diagnosis of anorexia
- Known osteoporosis
- Known refractory thrombocytopenia in subjects with acute leukemias
- Known or suspected allergy against triptorelin
- Direct radiation of the gonads previous or planned (TBI allowed)
- Mental inability, reluctance or language difficulties that result in difficulty understanding the meaning of study participation

6.3. Screening

All young women (age 18-42) and teenagers (age 14-17) will receive reproductive counseling and may elect to undergo fertility preservation through cryopreservation methods, according to the clinical situation, which is not an exclusion criterion for participation in the study. If all

inclusion criteria and no exclusion criteria are met, subjects will be enrolled and randomized in the study.

6.4. Withdrawal criteria

Subjects can discontinue their participation in the study at any time without any consequence to her continued treatment. The principal investigator/sponsor can at any time terminate the study for a subject due to, e.g., unacceptable adverse events/adverse reactions or because the subject does not follow procedures in the study protocol. If the subject discontinues the study, follow-up of this subject will be performed according to the clinic's routine.

Specific reasons for discontinuing a subject from further assessments are:

- Withdrawal of informed consent
- Not fulfilling the inclusion/exclusion criteria as stated above (incorrectly included)
- If the investigator considers it not to be in the subject´s best interest to continue participation in the study (e.g. due to non-tolerable adverse reactions or serious adverse events (SAEs))
- If there is significant protocol deviation
- If a concomitant therapy or surgery is reported or required which is likely to interfere with the results of the study or compromise subject safety such as; recurring cancer treated with IR or gonadotoxic cytostatics, oophorectomy, introduction of interfering hormonal treatments or additional treatments with the study drug
- Decision made by authorities that the subject should be discontinued

A discontinued subject should, if possible, be asked to come to the clinic for a final assessment.

The reason for subject discontinuation will be documented in the eCRF. If a subject is discontinued due to an SAE, the nature of the event and its clinical course must be fully documented. The investigator must follow-up the subject until the SAE is resolved, becomes clinically insignificant, or is stabilized. The sponsor has the right to keep data collected up until the subject discontinues study participation. A study subject who has been randomized and who is withdrawn from the study will not be included again and the subject identification number not reused.

7. Study treatments

7.1. Description of investigational product(s)

Active agent: Triptorelin embonate equivalent to triptorelin
Trade name: Pamorelin®
Manufacturer: Institut Produits Synthèse (IPSEN) AB
Formulation: Powder and solvent for prolonged-release suspension for injection. The powder is white to off-white powder and the solvent is a clear solution.
Storage instructions: Do not store above 25°C. The shelf life of triptorelin is 3 years. Use immediately after reconstitution. From a microbiological point of view, the product should be used immediately. If not used immediately, storage times and conditions are the responsibility of the user and should normally not exceed 24 hours at 15°C to 25°C.

Placebo: 0.9% Sodium Chloride solution containing 9 mg/ml sodium chloride in water for injection.
Study drug preparation: Triptorelin and placebo for administration must be prepared by an unblinded research nurse at each site. Triptorelin and placebo syringes need to be covered with aluminium foil by the unblinded research nurse to maintain the blind, thereafter another research nurse, blinded to the type of study drug, will give the injection.
Triptorelin: The suspension for injection must be reconstituted using an aseptic technique and only using the ampoule of solvent for injection. The instruction for reconstitution hereafter and in the leaflet must be strictly followed. The solvent should be drawn into the syringe provided using the reconstitution needle (20 G, without safety device) and transferred to the vial containing the powder. The vial should be reconstituted by swirling the vial gently from side to side for long enough until a homogenous, milky suspension is formed. Do not invert the vial. It is important to check there is no unsuspended powder in the vial.

The suspension obtained should then be drawn back into the syringe, without inverting the vial. The reconstitution needle should then be changed and the injection needle (20 G, with safety device) used to administer the product.

As the product is a suspension, the injection should be administered immediately after reconstitution to prevent precipitation. For single use only. Used injections, used needles, any unused suspension or other waste material should be disposed of in accordance with local requirements.

Triptorelin and placebo: To maintain the study blind, triptorelin and placebo syringes will be covered with aluminium foil by the unblinded research nurse that prepares the syringe, and the injection to study subjects will be performed by the blinded research nurse at each site. The blinded research nurse also will handle the data entry into the eCRF and keep essential log forms updated.

7.2. Dose and administration

Two different doses of triptorelin will be supplied for this study.

Triptorelin: 11.25 mg will be given for subjects having at least 3 months gonadotoxic treatment; one injection of 11.25 mg will compensate for 3 months' effect of the study drug. 1 vial contains 11.25 mg triptorelin embonate equivalent to 11.25 mg triptorelin. 1 ml reconstituted suspension contains 5.625 mg triptorelin after dissolution in 2 ml solvent.

Triptorelin: 3.75 mg will be given during one-month of gonadotoxic treatment; one injection of 3.75 mg will compensate for 1 month' effect of the study drug. 1 vial contains 3.75 mg triptorelin embonate equivalent to 3.75 mg triptorelin. 1 ml reconstituted suspension contains 1.875 mg triptorelin after dissolution in 2 ml solvent.

Study subjects will receive a combination of 11.25 mg (3 months' effect) and 3.75 mg (1 month' effect) of triptorelin/placebo (see example below).

Make sure that the study treatment covers the full duration of gonadotoxic treatment until clearance of the gonadotoxic drug. The estimated time of study treatment in table 3 and 4 is calculated from disease-specific treatment start to the end of the last disease-specific treatment block including gonadotoxic agents. *Take special notice if the first dose of study drug is administered before the start of disease-specific cytostatic treatment.*

Placebo: will be provided both as one injection compensating for 3 months' effect and one injection compensating for 1 month' effect to maintain the study blind.

Example:
Subjects treated for lymphoma receiving 6 months of gonadotoxic treatment:

- 3 injections of 3.75 mg triptorelin/placebo and 1 injection of 11.25 mg triptorelin/placebo
    1. 1 injection of 3.75 mg triptorelin/placebo Month 1/Day 1
    2. 1 injection of 3.75 mg triptorelin/placebo Month 2/Day 1
    3. 1 injection of 3.75 mg triptorelin/placebo Month 3/Day 1
    4. 1 injection of 11.25 mg triptorelin/placebo Month 4/Day 1

Subjects treated for lymphoblast lymphoma receiving 5 months of gonadotoxic treatment:

- 2 injections of 3.75 mg triptorelin/placebo and 1 injection of 11.25 mg triptorelin/placebo
    1. 1 injection of 3.75 mg triptorelin/placebo Month 1/Day 1
    2. 1 injection of 3.75 mg triptorelin/placebo Month 2/Day 1
    3. 1 injection of 11.25 mg triptorelin/placebo Month 3/Day 1

Route of administration: Triptorelin/placebo will be injected intramuscularly. The injection site should be varied periodically. Since triptorelin is a suspension of microgranules, inadvertent intravascular injection must be strictly avoided.
Duration: Each subject will take the IMP as long as the gonadotoxic treatment is lasting.
Dose change/reduction: No change and no reduction of the two different triptorelin doses are allowed.

In case of a SAE with possible relation to the treatment with triptorelin can be necessary to unblind the study subject.

7.3. Packaging, labelling, and handling of investigational products(s)

Packaging: 6 ml (type I glass) with bromobutyl stopper and aluminium flip-off cap. Ampoule (type I glass) containing 2 ml of sterile solvent for suspension.
Box of:

- 1 vial, 1 ampoule and 1 blister containing 1 injection syringe and 2 injection needles

Triptorelin will be labelled and packaged by Tamro, Gothenburg, Sweden. The labelling and packaging will be done according to local requirements and Good Manufacturing Practice. Delivery of triptorelin will be done by Tamro directly to each site. Triptorelin has to be stored in original package at room temperature (15°C - 25°C). Upon delivery of triptorelin to site, the dispatched temperature data loggers should be assessed for any excursions. Excursions have to be documented and reported to the sponsor immediately; according to sponsor's decision affected triptorelin should be destroyed by site but only after reconciliation by monitor and replaced by another shipment.

After reconstitution of triptorelin at site, triptorelin should normally not exceed 24 hours at 15°C - 25°C. Any excursion after reconstitution at site need to be reported to the sponsor immediately; according to sponsor's decision affected, reconstituted triptorelin should be destroyed by site but only after reconciliation by monitor and replaced by the same type of drug from shelf at site.

Excursions that occur during storage and reconstitution of triptorelin, have to be reported to the monitor immediately and subsequently to the sponsor, if significant. For further information please refer to the summary of product characteristics of triptorelin.

7.4. Drug accountability and treatment compliance

The investigator is responsible for ensuring triptorelin/placebo accountability, including reconciliation of study drugs and maintenance of study drug records.

- Upon receipt of triptorelin, site will check for accurate delivery and acknowledge receipt by signing and dating the documentation provided by delivery company. A copy of each document will be filed in the Trial Master File and another copy will be retained for the Investigator Site File.
- The dispensing and reconstitution of blinded triptorelin/placebo from site will be carefully recorded on the appropriate drug accountability forms and an accurate accounting will be available for verification by the monitor at each monitoring visit:
- The investigator should maintain logs/records which adequately document that:
    - The triptorelin/placebo were provided to the subjects in the doses specified by the protocol/amendment(s) and randomization
- All triptorelin provided by delivery company were fully reconciled

Triptorelin/placebo that have been dispensed to a subject are not allowed to be re-dispensed to a different subject. Unused triptorelin/placebo must not be discarded or used for any purpose other than the present study. Triptorelin/placebo and boxes, vials and syringes should be destroyed by site after reconciliation by the monitor. The monitor will periodically control the triptorelin/placebo accountability log forms and check all blinded syringes used by the subjects at site.

7.5. Randomization

At the time of inclusion, inclusion and exclusion criteria are entered into the randomization application, which is a web-based instrument (ALEA) and will be run and making use of the Clinical Trials Office (CTO), Center for Clinical Cancer Studies at Karolinska University Hospital. Username and password are required to log in; each personnel authorized to register subjects has a personal login username and password. If all criteria are met, subjects are randomized to receive triptorelin or placebo in a 1:1 ratio. At randomization, the ALEA will assign a unique subject identification number and the randomization arm to each subject. The subject's identification number will be used on all study related documents including the eCRF. A single subject cannot be assigned more than one identification number. An email confirmation of the randomization will immediately be returned to site together with the subject unique identification number. This confirmation will be considered as source document.

Subjects will be stratified per diagnosis (breast cancer, lymphoma, sarcoma, leukemia) and for subjects with breast cancer also for age groups (14-34; ≥35). Randomization is based on permutated block technique.

Subjects are randomized consecutively as they are found to be eligible for inclusion in the study. If a subject actively discontinues their study participation, their subject code will not be reused, and the subject will not be allowed to re-enter the study again. All subjects should commence study treatment within -14 and +7 days of initiation of disease-specific

chemotherapy (except for the HR ALL and the AML subjects who will be eligeble for inclusion when they are assigned SCT).

7.6. Blinding

This is a randomized, double-blinded study. Subjects will receive triptorelin or placebo. All personnel involved in the study, sponsor, investigators, site staffs and monitors, statisticians and subjects, except personnel preparing triptorelin/placebo at the local site and an unblinded monitor, will be blinded during the study. Unblinded research nurse at each site will prepare blinded triptorelin/placebo to subjects and the web-based randomization system ALEA will be used to allocate of blinded triptorelin/placebo to subjects at randomization.

The following precautions are to be taken to ensure that blinding is adequately maintained throughout the study.

7.7. Code breaking

Investigators will not be provided with randomization codes. The codes will be maintained within the web-based randomization system ALEA, which has the functionality to allow the investigator and responsible person for pharmacovigilance at CTO to break the blind for an individual subject in case of emergency.

When a SAE is judged reportable on an expedited basis, the blind will be broken only for that specific subject by the pharmacovigilance responsible person at CTO even if the investigator has not broken the blind. Blinded site staff (including investigator at a site) will have no direct access to the ALEA randomization program as the information in the randomization system contains study treatment information and can unblind the blinded team. It is recommended that the investigator contact the sponsor if possible, to discuss the particular situation before breaking the blind.

All efficacy and safety analyses will be performed after the study is completed and the study database is released for unblinding. Data will be unblinded for the statisticians only and analyzed according to the locked and signed Statistical Analyses Plan (SAP) when all subjects finalized the 12 months follow-up period. At the end of study, after the extended follow-up period of 5 years when all study data for the mentioned visits are collected and verified in the eCRF system, the unblinding of all subjects will take place for investigators and sponsor.

7.8. Concomitant medications

Medications that are considered necessary for the safety and well-being of the subject can be given at the discretion of the investigator, unless otherwise specified as an exclusion criterion. Concomitant medications deviating from treatment protocol (study treatment and cancer treatment) should be reported in the eCRF.

7.9. Destruction

Both redundant and reconstituted triptorelin and placebo will be destroyed on site.

7.10. Treatment after study end

The study subjects will receive no further treatment with the study drug after the initial treatment period but most included subjects will proceed with cancer treatment according to their assigned protocol after the end of study treatment. The study does not interfer with the clinical protocols for cancer treatment.

Rodriguez-Wallberg KA, *et al. BMJ Open* 2023; 13:e078023. doi: 10.1136/bmjopen-2023-078023

8. Assessment of efficacy and safety

8.1. Assessment of clinical efficacy

The main outcome of this study will be set to changes in the AMH, the currently most reliable biochemical marker of ovarian reserve (Kelsey et al, 2011; Dewailly et al, 2014), which does not fluctuate during the menstrual cycle.

The fully automated AMH assay developed on the Elecsys/Cobas electrochemiluminescence immunoassay platform currently available at Karolinska University Hospital laboratory will be used (Gassner D, 2014).

Additional hormonal/biochemical markers such as Inhibin and indicators of ovarian failure such as FSH, will be followed, as well as estradiol estimated by ultra-sensitive assay, also available at the clinical laboratory of Karolinska university hospital. The FSH has been clinically used over the years as a marker of ovarian insufficiency, i.e., to establish the diagnosis of ovarian failure or premature menopause, as it correlates with the depletion of follicles in the ovaries and the resistance of the ovaries to gonadotropins, with a reduction in both estrogen and inhibin secretion (Honour JW, 2017).

In our study, the blood samples for hormonal/biochemical marker determinations will be taken on cycle day 3, as standardized in menstruating women and followed-up according to the schematic Figure 3. Because it takes approximately 4-6 months for surviving primordial follicles to grow and produce hormones that result in menstruation after chemotherapy, any benefit from GnRHa would probably be reflected in the 6- to 12-month hormone assessment and menstruation assessment (Turan et al, 2019).

Secondary (clinical) endpoints for estimation of the ovarian reserve will include the count of AFC by ultrasound, menstrual resumption, cycle regularity and cycle length. Because it takes approximately 4-6 months for surviving primordial follicles to grow and produce hormones that result in menstruation after a chemotherapy insult, any benefit from GnRHa would be reflected in the 6- to 12-month menstruation assessment (Turan et al, 2019).

Fertility and childbirth (also secondary endpoints for the study) after cancer treatment will be investigated in the women who attempt pregnancy during follow-up. The reproductive outcome data will be collected during follow-up medical visits, which will be standardized also by using a checklist and a specifically developed validated questionnaire for this project that will include clinical data and data on reproductive health, sexuality and quality of health aspects, pregnancy wish, pregnancy attempts and pregnancy outcomes.

Furthermore, as secondary endpoint, quality of life parameters during chemotherapy and after cancer treatment through standardized validated formularies, development of co-morbidities during follow-up.

Oncological outcomes including recurrence rate, overall survival, relapse-free survival and co-morbidities will also be estimated.

8.1.1. Primary variable

Levels of the primary variable, AMH in women with breast cancer, will be measured at baseline, at EoT and continuously during follow up 6, 12, months and years 2, 3, 4, 5 after EoT.

8.1.2. Secondary variable(s)

Levels of the secondary variable, AMH in women with lymphomas, sarcomas and leukemias, will be measured at baseline, at EoT and continuously during follow up 6, 12, months and years 2, 3, 4, 5 after EoT.

The secondary variable, AFC and ovarian blood flow, will be estimated at baseline, at EoT and continuously at follow-up 6, 12, months and years 2, 3, 4, 5 after EoT.

Additional secondary variables, the hormone/biochemical markers e.g., FSH, inhibin and estradiol will be measured at baseline, at EoT and continuously at follow-up 6, 12, months and years 2, 3, 4, 5 after EoT.

During follow-up, all young women (age 18-42) and teenagers (age 14-17) will be followed for 5-years after completed gonadotoxic treatment (clinical follow-up and biochemical markers of ovarian reserve), within the frame of this study (Figure 3).

Menstruation data and quality of life parameters during chemotherapy and after cancer treatment will be investigated at baseline, at EoT and continuously at follow-up 6, 12, months and years 2, 3, 4, 5 after EoT.

Markers of bone mineral density will be measured at baseline and EoT, 1 and 5 years after EoT.

8.2. Assessment of clinical safety

The study drug is well established for other indications in Sweden and used for this indication in Sweden and globally. During the study we will measure safety blood samples at baseline and at EoT as well as access the safety blood markers taken during chemotheraphy through the subject´s medical records. Safety blood samples will assess routine blood parameters (a complete blood count, including B-hemoglobulin, WBC, differential count and platelets), liver status (ALT, AST, bilirubin, ALP, GGT), renal status (Sodium, Potassium, Calcium, Creatinine, Albumin). Vital parameters will be assessed during each study visit and a validated questionnaire regarding sexual function, quality of life and depression/anxiety will be answered continuously during the study period. We will also measure bone mineral density as a safety variable during the study at baseline, at EoT, and 12 months and 5 years follow-up, and ECG at baseline and at EoT. Inspection of injection site and measuring of blood pressure and heart rate will be performed at the screening/baseline visit and at every study visit throughout the study. Any adverse events will be logged in the eCRF.

9. Handling of Adverse Events

9.1. Definitions

9.1.1. Adverse Event (AE)

Adverse event (AE): Any untoward medical occurrence in a clinical investigation subject administered a medicinal product and, which does not necessarily have a causal relationship with the treatment, can be an unfavorable and unintended sign (including an abnormal laboratory discovery), symptom or disease temporally associated with the use of the medicinal (investigational) product, whether or not related to the medicinal (investigational) product.

9.1.2. Adverse Drug Reaction (ADR)

In the pre-approval clinical experience with a new medicinal product or new use of a medicinal product, and particularly as the therapeutic dose(s) may not be established, all noxious and unintended responses to the medicinal product related to any dose should be considered adverse drug reaction (ADR). The phrase "response" to a medicinal product means that the causal relationship between the medical product and an adverse event is at least a reasonable possibility, that is the relationship cannot be ruled out.

9.1.3. Serious Adverse Event (SAE)

Serious adverse event (SAE): Any untoward medical occurrence that at any dose:

- results in death
- is life-threatening
- requires insubject hospitalization or prolongation of existing hospitalization
- results in persistent or significant disability or incapacity
- results in a congenital anomaly/malformation

Medical and scientific assessment will be made to determine if an event is "serious" and whether it would prompt reporting in other situations, for example important medical events that may not be directly life-threatening or result in death or hospitalization but may compromise the study subject or may require intervention to prevent one of the other results set forth in the definitions above. These should also normally be considered as SAEs.

9.1.4. Suspected Unexpected Serious Adverse Reaction (SUSAR)

SUSAR: A reaction/event that is unexpected, serious, and suspected to be caused by the treatment, i.e. adverse events that are not included in the Investigator's Brochure (IB) or SPC.

9.2. Assessment of adverse events

9.2.1. Assessment of causal relationship

The investigator is responsible for determining whether there is a causal relationship between the AE/SAE and use of the investigational product.

Those AEs which are suspected of having a relationship to the investigational product will be followed up until the study subject has recovered or is well taken care of and on their way to good recovery (see also section 9.4, Follow-up Adverse Events).

All AE will be categorized either as likely related, possibly related, or not related, in accordance with the definitions below:

Likely related: Clinical event, including abnormal results from laboratory analyses, occurring within a reasonable time after administration of the intervention/investigational product. It is unlikely that the event can be attributed to underlying disease or other medications but is most likely caused by the investigational product and its emergence is reasonable in relationship with use of the investigational product.

Possibly related: Clinical event, including abnormal results from laboratory analyses, occurring within a reasonable time after administration of the intervention/investigational product. The event could be explained by the investigational product and its emergence is reasonable in relationship with use of the investigational product, but there is insufficient information to determine the relationship. The event could be explained by an underlying disease or other medications.

Not related: Clinical event, including abnormal results from laboratory analyses, that is not reasonably related to the use of the intervention/investigational product. The event is unlikely related to the intervention/investigational product and can be explained by other medications or underlying disease.

9.2.2. Assessment of severity

Each adverse event shall be classified by an investigator as mild, moderate or severe.

Mild: The adverse event is relatively tolerable and transient in nature but does not affect the study subject's normal life.

Moderate: The adverse event causes deterioration of function but does not affect health. The event can be sufficiently unpleasant and interferes with normal activities but does not completely obstruct them.

Severe: The adverse event causes deterioration of function or work ability or poses a health risk to the study subject.

9.3. Reporting and registration of adverse events

At each study visit, adverse events (AE) related to the study product are registered, starting at study randomization and up to 90 days after the last dose of the investigational products, triptorelin and placebo (sodium chloride). All AE that occur during the study and which are observed by the investigator/study nurse or reported by the subject will be registered in the eCRF regardless of whether they are related to the investigational products triptorelin and placebo (sodium chloride) or not.

Assessment of causal relationship, severity, and whether the AE is considered to be an SAE or not will be done by the investigator directly on study-specific worksheet. At minimum, for each AE/SAE, a description of the event is recorded (diagnosis/symptom if diagnosis is missing), start and stop dates, causal relationship, severity, if the AE is considered to be an SAE or not, measures and outcome.

The following adverse reactions in women, considered as at least possibly related to triptorelin were reported as:

Very common (≥1/10): Libido decreased, mood disorder, sleep disorder (including insomnia), headache, hot flushes, acne, hyperhidrosis, seborrhea, breast disorder, dyspareunia, genital

bleeding (including vaginal bleeding, withdrawal bleed), ovarian hyperstimulation syndrome, ovarian hypertrophy, pelvic pain, vulvovaginal dryness and asthenia.

Common (≥1/100 to <1/10): Hypersensitivity, depression, nervousness, dizziness, nausea, abdominal pain, abdominal discomfort, arthralgia, muscle spasms, pain in the extremities, breast pain, injection site reaction (including pain, swelling, erythema and inflammation), oedema peripheral, tiredness and weight increased.

Uncommon (≥1/1000 to <1/100): Loss of appetite, fluid retention, affect lability, anxiety, depression, disorientation, dysgeusia, hypoesthesia, syncope, memory impairment, disturbance in attention, paraesthesia, tremor, dry eye, visual impairment, vertigo, palpitations, dyspnea, epistaxis, abdominal distension, dry mouth, flatulence, mouth ulceration, vomiting, alopecia, dry skin, hirsutism, onychoclasis, pruritus, rash, back pain, myalgia, coital bleeding, cystocele, menstrual disorder (including dysmenorrhea, metrorrhagia and menorrhagia), ovarian cyst, vaginal discharge and weight decreased.

Additional post-marketing AEs (frequency not known): Anaphylactic shock, confusional state, visual disturbance, hypertension, diarrhea, angioneurotic oedema, urticaria, muscular weakness, amenorrhea, pyrexia, malaise, blood alkaline phosphatase increased and blood pressure increased.

The following adverse reactions in women with breast cancer receiving triptorelin in combination with tamoxifen or exemestane, were reported as:

Very common (≥1/10): Nausea, fatigue, musculoskeletal disorder, osteoporosis, insomnia, libido decreased, depression, urinary incontinence, dyspareunia, vulvovaginal dryness, hyperhidrosis, hot flush and hypertension.

Common (≥1/100 to <1/10): Diabetes mellitus (glucose intolerance), hyperglycemia, injection site reaction, hypersensitivity, fracture and embolism.

Uncommon (≥1/1000 to <1/100): Myocardial ischemia, cerebral ischemia and central nervous system hemorrhage.

Rare (≥1/10000 to <1/1000): QT prolongation.

The following adverse reactions in children, considered as at least possibly related to triptorelin were reported as:

Very common (≥1/10): Vaginal bleeding (including hemorrhage, withdrawal bleed, uterine hemorrhage, vaginal discharge, vaginal bleeding including spotting).

Common (≥1/100 to <1/10): Hypersensitivity, headache, hot flushes, abdominal pain, acne, injection site reaction (including pain, erythema and inflammation) and weight increased.

Uncommon (≥1/1000 to <1/100): Obesity, mood altered, vision impairment, epistaxis, vomiting, constipation, nausea, pruritus, rash, urticaria, neck pain, breast pain and malaise.

Additional post-marketing AEs (frequency not known): Anaphylactic shock, affect lability, depression, nervousness, visual disturbance, hypertension, angioneurotic oedema, myalgia, blood prolactin increased and blood pressure increased.

The following symptoms are clearly related to the process and the expected course of condition and therefore will not be reported as AE:

- All AE related to the chemotherapy of the underlying disease, such as breast cancer, acute leukemias, lymphomas and sarcomas

9.3.1. Reporting of adverse events (AE)

All AE will be registered on a separate AE form in the eCRF with complete information regarding seriousness, intensity and causality, and all AE assessments will be made by an investigator.

9.3.2. Reporting of serious adverse events (SAE)

Serious adverse events (SAE) will be reported by the investigator to the sponsor on a separate SAE form within 24 hours of the investigator being informed of the SAE. Follow-up information describing the outcome and handling of the SAE will be reported as soon available. The original SAE form must be filed in the Investigator Site File.

All SAE should be reported within 24 hours after notification of the study personel to the Clinical Trial Office (CTO) at the Center for Clinical Cancer Studies (CKC), Cancer Theme, Karolinska University Hospital, Stockholm, by email to:

ctosafety.karolinska@regionstockholm.se

Based on knowledge of the disease in question and expected clinical course, some events that are otherwise serious are not considered as SAEs in this study. The following is a list of SAEs that shall not be reported as SAEs:

- All SAE related to the chemotherapy of the underlying disease, such as breast cancer, acute leukemias, lymphomas and sarcomas
- If a subject is hospitalized with a documented cancer-related problem, this will not be reported as an SAE
- Pregnancy and childbirth except from the period of the disease-specific chemotherapy

9.3.3. Reporting of Suspected Unexpected Serious Adverse Reactions (SUSAR)

Those SAE which are assessed by sponsor to be SUSAR are reported via a CIOMS form to the European Medicines Agency (EudraVigilance database) according to the specified time frames. Since the sponsor is non-commercial and the study is investigator-initiated study, the Medical Products Agency (in Swedish: Läkemedelsverket) will be requested to provide help to report the SUSAR to the EudraVigilance database. The CIOMS form will be used for submitting the report to the Medical Products Agency.

SUSAR that are fatal or life-threatening are reported as soon as possible and no later than 7 days after the incident has become known to the sponsor. Relevant follow-up information is sent thereafter within an additional 8 days. Other SUSAR are reported as soon as possible and no later than 15 days after they have come to the sponsor’s knowledge.

Information about SUSAR occurring during the study is compiled by the sponsor and sent out to the principal investigator at all participating centers. In order to preserve the integrity of the study, reporting of SUSAR to investigators will be done without unblinding, that is, without specifying which investigational product (triptorelin or placebo) the study subject received.

9.4. Follow-up of Adverse Events

If an AE has been reported for a subject, the nature of the event and its clinical course must be fully documented. The investigator must follow-up the subject until the AE is resolved, becomes clinically insignificant, or is stabilized.

9.5. Annual Safety Report (Development Safety Update Report, DSUR)

While the study is ongoing, a Development Safety Update Report (DSUR) will be reported annually to the MPA by the sponsor.

9.6. Procedures in case of emergencies, overdose or pregnancy

If a study subject who participates in a clinical trial for investigational products becomes pregnant, in this study, except from the period of follow-up when pregnancy and childbirth are diserable, this person must be followed up until the birth has taken place. If the fetus/child has any congenital malformation, this must be reported as a SAE.

9.7. Reference Safety Information

Reference safety information for assessment of an AE/SAE as expected or unexpected can be found in the Summary of Product Characteristics (SPC) for Pamorelin.

10. Statistics

The principal features of the statistical analysis to be performed are described in this section. A more technical and detailed elaboration of the principal features will be presented in a separate Statistical Analysis Plan (SAP).

10.1. Statistical analysis plan and database locks

The SAP contains a detailed description of all statistical methodology utilized. The final SAP will take into account any amendment to the protocol.

A SAP version 1.0 will be finalized and signed before all subjects finalized the 12 months follow-up period. An interim database lock (soft database lock) will then be performed, when all subjects finalized the 12 months follow-up period. When the study is completed a Clean File Report will be written and signed including decision on analysis sets. Final database lock can then be performed. Before the Clean File an updated SAP can be written and signed which incorporates updates compatered to SAP version 1.0.

10.2. Analysis population

This study uses three analysis sets, the Intention-to-treat (ITT), the per protocol analysis set (PP), and the safety analysis set. The classification of each subject with respect to each analysis set will be done prior to respective database lock.

Intention-to-treat (ITT) will be all randomized subjects who met all inclusion criteria and none of the exclusion criteria.

In PP, only the randomized subjects who met all inclusion criteria and none of the exclusion criteria without major protocol deviations will be included. Non-adherence to the study treatment will lead to exclusion from PP.

Final criteria for defining PP will be defined in the SAP.

The safety population includes all randomized subjects who receive study product.

10.3. Method of statistical analyses

This section describes the statistical analysis as it is foreseen at the time of planning the study. The statistical analyses will be further detailed in the SAP.

10.3.1. General Principles

Continuous variables will be summarized as number of subjects (n), mean, median, standard deviation, Quartile 1 (Q1) and Quartile 3 (Q3 and range (min, max) by visit. The change from baseline at each respective visit will also be presented. Discrete (categorical/ordinal) variables will be summarized in frequency tables (frequency and proportion) by visit.

Graphical presentations will be used as appropriate.

All statistical tests will be two-sided on significance level 5 %.

All statistical analyses will be performed using SAS® (Version 9.4 or higher, SAS Institute Inc., Cary, NC, USA) unless otherwise stated.

10.3.2. Primary and secondary analyses

All endpoints will be summarised descriptively. The results will in general be presented according to visit and treatment group.

The statistical analysis of the primary endpoint will be performed using the $\log_{10}$-transformation of AMH levels. However, the descriptive summaries will use untransformed AMH-levels for ease of interpretation. The primary endpoint (ovarian reserve measured with AMH in women with breast cancer) will be analyzed using an ANCOVA -model. The model will include treatment group, age, ovarian reserve (AMH) at baseline, inclusion site and type of cancer treatment as covariates (fixed effects). The outcome variable will be recovery after 12 months from EoT in AMH level (recovery is defined as the change between 12 months after EoTcompared to at EoT). Model-based estimates (LSMeans) of the difference between GnRHa group and placebo group with respect to recovery in AMH level at 12 months after EoT along with a two-sided 95 % confidence interval (CI) and p-value, will be provided.

Main analysis for the primary endpoint will be done using the ITT population. Analysis on the PP population will be performed as sensitivity analysis.

If assumptions underlying the primary analysis are not fulfilled a non-parametric analysis will be used instead.

Key secondary endpoint, recovery in AMH-levels after 12 months from EoT in women with lymphomas, sarcomas and leukemias, will be analysed using the same methods as the primary endpoint.

AMH levels over time since EoT will be presented in figures by treatment group.

Secondary endpoints, AMH change from baseline, AFC and FSH, inhibin and estradiol using a Mixed Model Repeated Measures (MMRM) with the same covariates as in the model for the primary endpoint.

Analyses and methods for presenting the other secondary endpoints will be described in the SAP.

Descriptive statistics will be used to present the safety outcomes in the two treatment groups as following the general principles given above, including incidence and severity of AEs and SAEs and incidence of AEs leading to investigation withdrawal.

The number of events, as well as the number and percentage of subjects reporting any AE will be summarised by Preferred Term (PT) and System Organ Class (SOC).

10.3.3. Handling of missing data

All available data will be used in the statistical analysis. A subject who withdraws prior to the last planned visit in the study will be included in the analyses up to the time of discontinuation.
Some of the analyses uses a MMRM model which handle missing data but assumes Missing At Random (MAR).

10.3.4. Subgroup analysis

Subgroup analysis will be performed on ITT population for AMH change on these prospectively defined subgroups:

- Age (14-19; 20-34; ≥35)
- Ovarian reserve (AMH) at baseline (0.68-2.3; 2.4-4.9; ≥5 (microgr/L))
- Cancer diagnosis (breast cancer, lymphoma, sarcoma, leukemia)
- Type of cancer treatment (high risk, intermediade risk, low risk)
- Exposure to gonadotoxic cytostatics (corresponding to cumulative dose, mg CPA)

Complementary subgroup analyses may be suggested in the SAP.

10.4. Adjustment of significance and confidence interval

There is only one primary endpoint and therefore no adjustment for multiple comparisons will be performed.

For the secondary endpoints, no adjustment for multiple comparisons will be made, and it should therefore be noted that if the null hypothesis is true the probability of making a type I error increases with the number of statistical tests performed. Therefore, some care should be taken when interpreting the results.

10.5. Sample size calculations

The sample size calculation used log_{10}-transformed AMH levels (with a ratio 1:1 between the treatment groups). In Goldfarb et al. (2021) subjects in treatment groups AC-based and CMF had on average an AMH-level of around 0.3 12 months after EoT. Assuming further that the GnRHa group would have average AMH-level around 0.5 12 months after EoT (where the difference 0.5-0.3=0.2 constitutes a clinical relevant difference). Then using log_{10}-transformation the difference between the GnRHa group and the control group from EoT to EoT + 12 months is around 0.22.

It was further assumed that data are approximately normally distributed, and that each treatment group has a standard deviation (SD) of 0.63. The SD is the average value of SD in AC-based and CMF treatment groups in Goldfarb et al. (2021) at 12 months after EoT. Under these assumptions, the hypothesis test (a two-sided test with significance level 5 %) of the mean effect in the active group vs the control group will have a power of at least 80% if the sample size is 300 (including an assumed 15% drop-out rate). The assumptions for the AMH levels are based on Goldfarb et al. (2021).

The sample size calculation above was done for the primary endpoint where subjects with breast cancer are included. The study will also recruit subjects with lymphomas, acute leukemias and sarcomas. All these subject groups are likely to be smaller and therefore the study is not powered to show a difference in AMH levels between GnRHa and placebo groups within any of these subject groups.

Sample size calculations were performed using SAS® (version 9.4, SAS Institute Inc., Cary, NC, USA).

10.6. Interim report

An interim report will be produced after last enrolled subject has completed follow up 12 months after EoT (primary endpoint). A statistical interim analysis is not planned. The interim report will include analyses of the primary and some secondary endpoints based on data assembled for each enrolled subject up until the time point corresponding to 12 months after EoT. The statistical analyses and desriptive summaries included in the interim report will be specified in the predefined (locked) SAP ver 1.0, see Section 10.1. The database assembled data will be locked and the study unblinded only for the statiticians. However, analyses results and aggregated descriptive summaries may be disclosed to the sponsor and published. The study can be terminated if the enrollment process cannot be expected to be completed within a reasonable time frame.

11. Quality Control and Quality Assurance

11.1. Training of Study Personnel

It is the responsibility of the principal investigator to ensure that all personnel involved in the study are fully informed of all relevant aspects of the study, including detailed knowledge of and training in all procedures to be followed. All co-investigators and other responsible personnel should be listed together with their function in the study on a delegation and signature log. This log should be continuously updated, signed by the principal investigator and stored in the Investigator Site File. The process of/tasks associated with enrollment, informed consent and follow-up of AEs must be performed by a physician and cannot be delegated to other study personnel, i.e. a study nurse, in a clinical drug trial.

11.2. Monitoring

The sponsor will appoint an independent monitor for quality control of the study. Monitoring will be performed before, during and after study completion in accordance with the ICH GCP guidelines and applicable ethical and regulatory requirements. The extent of monitoring will be described in a monitoring plan, which will be approved by the sponsor. Study conduct, source data, drug accountability, adherence to study protocol, GCP and regulatory requirements will be monitored.

A monitor from the Clinical Trial Office (CTO) at Center for Clinical Cancer Studies (CKC), Cancer Theme, Karolinska University Hospital will be responsible for coordinating the monitoring activities of the study and ensure adherence to ICH GCP guidelines. Before study initiation, the study protocol and CRF will be reviewed with the investigator and study personnel. The monitor will visit the site during the ongoing study in order to verify adherence to the clinical study protocol, completeness and accuracy of data entered in the CRF by comparing them with the source documents, and ensure that the subject's rights, safety, and well-being are met. The monitor will review the progress of the clinical study, and review CRF and original source data/documents with the study personnel, for accuracy of data recording, and facilities used in the clinical study. Visits will be documented in a monitoring log in the Investigator Site File and a monitoring report for each visit will be sent to the principal investigator and the sponsor.

Monitoring visits will be arranged in advance with study personnel. The investigator or study nurse and other responsible study personnel must be available during the monitoring visits and should prepare appropriate study material such as source data documents for the monitor.

11.3. Source data

The requirements regarding information in the medical records adhere to the Patient Data Act (SFS 2008:355) and "The Medical Product Agency's regulations on clinical trials of medicinal products for human use" (LVFS 2011:19), which means that except for information of importance for the wellbeing and care of the subject, the following minimum study specific information must be recorded:

- Study title and a brief description of the study in terms of treatment, dosing and assessments
- Date when subject information was given and when signed informed consent was obtained
- Study subject ID number
- Medically responsible study doctor, with contact details

The investigator must keep source documents for each subject in the study. For details and information that is study specific and of no interest for the medical care of the subject, CRF and other documents may be considered as source data. Prior to study start the expected source location of source data (e.g. medical record, laboratory reports, validated questionnaires and the eCRF), must be identified and documented. This will be done by completing a site-specific source data list. This list defines what documents contain the source data for each study specific parameter. The monitor will have access to medical records and source data after a secrecy agreement has been signed by the responsible party at the site as

well as by the monitor. The investigator must ensure that all source documents are accessible for monitoring and other quality control activities.

11.4. Deviations or serious breaches

Serious breaches and deviations from the study protocol, GCP and other regulations that significantly and directly affects, or with high likelihood could affect, study subjects' integrity or safety, or the scientific value of the study, shall be immediately reported within 7 days (from knowledge) to the Medical Products Agency (MPA). It is the sponsor's responsibility to judge the consequences of deviations that have occurred, and thus also to decide whether the MPA should be informed.
Minor deviations that do not affect subjects' integrity or safety, nor significantly affect the study's scientific value, are documented in the study documentation of the principal investigator and the sponsor.

11.5. Audits and inspections

Authorized representatives for the sponsor and Competent Authorities (CA) may carry out audits or inspections at the study site, including source data verification. The investigator must ensure that all source documents are available for audits and inspections. The purpose of an audit or inspection is to systematically and independently review all study-related activities and documents, so as to determine whether these activities were performed, registered, analyzed and reported correctly according to protocol, Good Clinical Practice (GCP) and applicable regulations.

12. Ethics

12.1. Compliance to the protocol, GCP and regulations

This study is designed and shall implemented, executed and reported in accordance with the study protocol, principles of the International Conference on Harmonization Tripartite Guideline for Good Clinical Practice (ICH-GCP E6 (R2)), applicable local regulations (i.e. European Directive 2001/20/EC) and the ethical principles of the Guidelines of the World Medical Association (WMA) Declaration of Helsinki (as amended by the 64th WMA General Assembly, Fortaleza, Brazil, October 2013). This is to ensure the safety and integrity of the study subjects as well as the quality of the data collected. This study will not commence until a Clinical Trial Authorization, CTA, is obtained from the MPA.

12.2. Ethical review of the study

It is the responsibility of the coordinating investigator to obtain approval of the study protocol, the subject information and informed consent form from the Swedish Ethical Review Authority (Etikprövningsmyndigheten, EPM) before enrollment of subjects into the study. It is the responsibility of the sponsor to obtain approval for study conduct from the MPA. The sponsor is also responsible for submitting the ethical approval to the MPA. At study completion or if the study is prematurely terminated, the sponsor will notify the MPA and the EPM. All correspondence with the MPA and the EPM will be filed in accordance with ICH/GCP-requirements.

12.3. Procedure for obtaining informed consent

The principal investigator at each site shall ensure that the subject is given full and adequate oral and written information about the study, its purpose, any risks and benefits as well as

inclusion and exclusion criteria. Subjects must also be informed that they are free to discontinue their participation in the study at any time without having to provide a reason. Subjects should be given the opportunity to ask questions and be allowed time to consider the provided information. If the person chooses to participate, both the subject and the investigator shall sign the informed consent form. When the subject is a teenager, the informed consent of both parents (legal representatives of the teenager) must be obtained. A copy of the subject information as well as the informed consent form shall be provided to the subject. The subject's signed and dated informed consent must be obtained before performing any study-specific activity in the study. Each subject who participated in the study will be identified by a subject number on a subject identification list. The subject agrees that monitors and inspectors may have access to their medical records. If new information is added to the study, the subject has the right to reconsider whether she will continue her participation.

12.4. Data protection

If any part of the data is handled by any other organization, inside or outside the EU, appropriate agreements and/or other documentation will be established, to ensure that the data processing is performed in accordance with the provisions of the General Data Protection Regulation (GDPR) and other relevant legislation, before any data transfer takes place.

The content of the informed consent form complies with relevant integrity and data protection legislation. In the subject information and the informed consent form, the subject will be given complete information about how collection, use and publication of their study data will take place. The subject information and the informed consent form will explain how study data are stored to maintain confidentiality in accordance with national data legislation. All information processed by the sponsor will be pseudonymized (coded) and study subjects will be identified by a study ID.

The informed consent form will also explain that for verification of the data, authorized representatives of the sponsor, as well as relevant authority, may require access to parts of medical records or study records that are relevant to the study, including the subject's medical history.
All study subjects will be informed that any data collected until informed consent withdrawal/discontinuation may be used in the study data analysis.

12.5. Insurances

Study subjects are insured through the Swedish Patient Insurance (Patientskadeförsäkringen) and the Swedish Pharmaceutical Insurance (Läkemedelsförsäkringen).

13. Substantial changes to the study

Substantial changes to the signed study protocol are only possible through approved protocol amendments and by agreement from all responsible persons. Information on non-substantial changes should be clearly noted in the amended protocol.

In the event that substantial changes to the protocol (e.g., changing of the main objective, primary or secondary endpoints, method to measure the primary endpoint, changing of the investigational product or dosage) will be made during the course of the study, approval from the Swedish Ethical Review Authority (Etikprövningsmyndigheten, EPM) as well as the Swedish Medical Products Agency (Läkemedelsverket) shall be obtained before any changes

are implemented. A change that concerns a new site, new investigator or a new study subject information sheet shall only be approved by EPM.
Non-substantial changes will be recorded and later entered in documentation that is submitted, for example in any subsequent notifications of a substantial change or in connection with End of Trial reporting.

No changes to the study procedures shall be implemented without the mutual agreement between the principal investigator and the sponsor. It is the responsibility of the sponsor/coordinating investigator to submit substantial changes to the authorities.

14. Collection, handling, and archiving data

14.1. Subject Identification Log

It is the principal investigator's responsibility to keep a screening log of all subjects that were considered for enrollment even if they were not subsequently enrolled. This information is necessary to verify that the subject population was selected without bias. The reasons for non-eligibility are to be defined in terms of one or more of the eligibility criteria. A screening number will be allocated each subject considered for enrollment. The investigator must also file a subject identification log of all enrolled subjects, with a specific study identification number for each subject, which includes sufficient information to link records, i.e. the CRF and medical records. This log should be accessible only by authorized personnel and should be stored for possible future inspections/audits.

All data will be registered, managed, and stored in a manner that enables correct reporting, interpretation, and verification. The complete Trial Master File, as well as source documents, will be archived for at least 10 years after the study is completed. Source data in the medical records system is stored and archived in accordance with the respective hospital regulations.

14.2. Case Report Form

An electronic Case Report Form (eCRF) is used for data collection. The eCRF is provided in the web-based SAS system PheedIt. This database is provided by Region Stockholm and is registered at CTO, Center for Clinical Cancer Studies, Cancer Theme, Karolinska University Hospital, Stockholm, Sweden. The investigator must ensure that data is registered and any corrections in the eCRF are made as stated in the study protocol and in accordance with the instructions. The investigator must ensure that the registered data is correct, complete, and that reporting takes place according to the timelines that have been predefined and agreed. The investigator signs the completed eCRF. A copy of the completed eCRF will be archived at the study site.

14.3. Archiving

The investigator and sponsor shall keep records of essential documents of the study for at least 10 years after study completion. The documents will be archived in a readable format for potential future audit or inspection by the regulatory authorities and must be stored with restricted access. It is the principal investigator's obligation to store the documents where they are protected from unauthorized access.

15. Notification of study completion, reporting, and publication

The Swedish Medical Products Agency shall be informed of the study's completion at latest 90 days after study end, through submission of a ”Declaration of End of Trial Notification” form.

If the study is prematurely terminated, the form “Declaration of End of Trial Notification” should only be used if the reason concerns the study's safety. If the sponsor terminates an ongoing study, the concerned authorities must be informed as soon as possible, but no later than within 15 days.

Within 6 months (due to study involving teenagers) after the study is completed, the results shall be analyzed, a clinical study report with individual data shall be prepared, and the study results shall also be reported in the EudraCT database.

Authors of the publications are those who actively participate in processing of the study protocol, recruiting subjects, compiling the results and plan for the articles. First the steering committee determines who will be actively participating in the analyses of data and writing manuscripts of all scientific reports addressing the primary and secondary endpoints, defined in the protocol from this study. The steering committee decides who will be the 1st author of respective publications as well as the co-authors regarding publications referred to above. The steering committee consists of Kenny Rodriguez-Wallberg, Jonas Bergh and Per Ljungman at Karolinska University Hospital.

16. References

- Anderson RA, Wallace WH. Antimüllerian hormone, the assessment of the ovarian reserve, and the reproductive outcome of the young patient with cancer. Fertil Steril. 2013 May;99(6):1469-75.
- Bedaiwy, M.A., et al., Gonadotropin-releasing hormone analog cotreatment for preservation of ovarian function during gonadotoxic chemotherapy: a systematic review and meta-analysis. Fertil Steril, 2011. 95(3): p. 906-14.

- Behringer, K., et al., No protection of the ovarian follicle pool with the use of GnRH-analogues or oral contraceptives in young women treated with escalated BEACOPP for advanced-stage Hodgkin lymphoma. Final results of a phase II trial from the German Hodgkin Study Group. Ann Oncol, 2010. 21(10): 2052-60.7.

- Brougham MF, Crofton PM, Johnson EJ, Evans N, Anderson RA, Wallace WH Anti-Müllerian hormone is a marker of gonadotoxicity in pre- and postpubertal girls treated for cancer: a prospective study.J Clin Endocrinol Metab. 2012 Jun;97(6):2059-67. doi: 10.1210/jc.2011-3180. Epub 2012 Apr 3.

- Chapman, R.M. and S.B. Sutcliffe, Protection of ovarian function by oral contraceptives in women receiving chemotherapy for Hodgkin's disease. Blood, 1981. 58(4): p. 849-51.
- Del Mastro, L., et al., Effect of the gonadotropin-releasing hormone analogue triptorelin on the occurrence of chemotherapy-induced early menopause in premenopausal women with breast cancer: a randomized trial. JAMA, 2011. 306(3):269-76.

- Dolmans MM, Taylor HS, Rodriguez-Wallberg KA, Blumenfeld Z, Lambertini M, von Wolff M, Donnez J. Utility of gonadotropin-releasing hormone agonists for fertility

preservation in women receiving chemotherapy: pros and cons. Fertil Steril. 2020 Oct;114(4):725-738. doi: 10.1016/j.fertnstert.2020.08.011. PMID: 33040981.

- Demeestere I, Brice P, Peccatori FA et al. No Evidence for the Benefit of Gonadotropin-Releasing Hormone Agonist in Preserving Ovarian Function and Fertility in Lymphoma Survivors Treated With Chemotherapy: Final Long-Term Report of a Prospective Randomized Trial. J Clin Oncol. 2016 Aug 1;34(22):2568-74.

- Dewailly D, Andersen CY, Balen A, Broekmans F, Dilaver N, Fanchin R, Griesinger G, Kelsey TW, La Marca A, Lambalk C, Mason H, Nelson SM, Visser JA, Wallace WH, Anderson RA.The physiology and clinical utility of anti-Mullerian hormone in women. Hum Reprod Update. 2014 May-Jun;20(3):370-85.

- Elgindy et al. Protecting Ovaries During Chemotherapy Through Gonad Suppression: A Systematic Review and Meta-analysis. Obstet Gynecol. 2015 Jul;126(1):187-95.

- Fox, K., H. Scialla, and H. Moore, Preventing chemotherapy-related amenorrhea using leuprolide during adjuvant chemotherapy for early stage breast cancer. Proc Am Soc Clin Oncol, 2001. 21: p. 98a.

- Gerber, B., et al., Effect of Luteinizing Hormone-Releasing Hormone Agonist on Ovarian Function After Modern Adjuvant Breast Cancer Chemotherapy: The GBG 37 ZORO Study. J Clin Oncol, 2011. 29(17): 2334.

- Gassner D, Jung R. First fully automated immunoassay for anti-Müllerian hormone. Clin Chem Lab Med. 2014 Aug;52(8):1143-52. doi: 10.1515/cclm-2014-0022. PMID: 24622790.

- Goldfarb SB, Turan V, Bedoschi G, Taylan E, Abdo N, Cigler T, Bang H, Patil S, Dickler MN, Oktay KH. Impact of adjuvant chemotherapy or tamoxifen-alone on the ovarian reserve of young women with breast cancer. Breast Cancer Res Treat. 2021 Jan;185(1):165-173. doi: 10.1007/s10549-020-05933-7. Epub 2020 Sep 15. PMID: 32930927; PMCID: PMC7877450

- Honour JW. Biochemistry of the menopause. Ann Clin Biochem. 2018 Jan;55(1):18- 33.

- Kelsey TW, Wright P, Nelson SM, Anderson RA, Wallace WH. A validated model of serum anti-müllerian hormone from conception to menopause. PLoS One. 2011;6(7):e22024.

- Lambertini M, Moore HCF, Leonard RCF, Loibl S, Munster P, Bruzzone M, Boni L, Unger JM, Anderson RA, Mehta K, Minton S, Poggio F, Albain KS, Adamson DJA, Gerber B, Cripps A, Bertelli G, Seiler S, Ceppi M, Partridge AH, Del Mastro L. Gonadotropin-Releasing Hormone Agonists During Chemotherapy for Preservation of Ovarian Function and Fertility in Premenopausal Patients With Early Breast Cancer: A Systematic Review and Meta-Analysis of Individual Patient-Level Data. J Clin Oncol. 2018 Jul 1;36(19):1981-1990.

- Moore et al. Goserelin for ovarian protection during breast-cancer adjuvant chemotherapy. N Engl J Med. 2015;372(10):923-932.

- Munster P.N., et al., Randomized Trial Using Gonadotropin-Releasing Hormone Agonist Triptorelin for the Preservation of Ovarian Function During (Neo)Adjuvant Chemotherapy for Breast Cancer. J Clin Oncol, 2012:

- Nitzschke M et al. GnRH analogs do not protect ovaries from chemotherapy- induced ultrastructural injury in Hodgkin's lymphoma patients. Arch Gynecol Obstet. 2010 Jul;282(1):83-8.

- Oktay K, Briggs D, Gosden RG. Ontogeny of follicle-stimulating hormone receptor gene expression in isolated human ovarian follicles. J Clin Endocrinol Metab. 1997 Nov;82(11):3748-51.

- Oktay K, Rodriguez-Wallberg KA, Munster P. Ovarian protection during adjuvnt chemotherapy. N Engl J Med, 2015 (372)23: 2268-9.

- Oktay K, Harvey BE, Partridge AH, Quinn GP, Reinecke J, Taylor HS, Wallace WH, Wang ET, Loren AW.Fertility Preservation in Patients With Cancer: ASCO Clinical Practice Guideline Update. J Clin Oncol. 2018 Jul 1;36(19):1994-2001.

- Rodriguez-Wallberg KA, Oktay K. Fertility preservation and pregnancy in women with and without BRCA mutation-positive breast cancer. Oncologist. 2012;17(11):1409-17.

- Rodriguez-Wallberg K, Turan V, Munster P, Oktay K. Can ovarian suppression with gonadotropin-releasing hormone analogs (GnRHa) preserve fertility in cancer patients? Ann Oncol. 2016 Feb;27(2):257.

- Rodriguez-Wallberg KA, Marklund A, Lundberg F, Wikander I, Milenkovic M, Anastacio A, Sergouniotis F, Wånggren K, Ekengren J, Lind & Borgström B. A prospective study of women and girls undergoing fertility preservation due to oncologic and non-oncologic indications in Sweden – Trends in patients' choices and benefit of the chosen methods after long-term follow-up. Acta Obstetricia Gynecologica Scandinavica, 2019a May;98(5):604-615.

- Rodriguez-Wallberg KA, Borgström B, Petersen C, Thurin-Kjellberg A, Mörse H, Giwercman A, Jarfelt M. National guidelines and multilingual age-adapted patient brochures and videos as decision aids for fertility preservation for children and teenagers with cancer – A multidisciplinary effort to improve children's information and access to fertility preservation in Sweden. Acta Obstetricia Gynecologica Scandinavica, 2019b May;98(5):679-680.

- Turan V, Bedoschi G, Rodriguez-Wallberg K, Sonmezer M, Pacheco FS, Oktem O, Taylor H, Oktay K. Utility of Gonadotropin-Releasing Hormone Agonists for Fertility Preservation: Lack of Biologic Basis and the Need to Prioritize Proven Methods. Journal of Clinical Oncology 2019 Jan 1;37(1):84-86.

- Wallace WH, Kelsey TW. Human ovarian reserve from conception to the menopause. PLoS One. 2010 Jan 27;5(1):e8772
